# Supplementary material for: Cross-tissue comparison of telomere length and quality metrics of DNA among individuals aged 8 to 70 years
Source: PLoS One. 2024 Feb 22;19(2):e0290918. doi: 10.1371/journal.pone.0290918 (PMC10883573; doi:10.1371/journal.pone.0290918)
Supplement: S1 Table — (PDF) [file pone.0290918.s001.pdf]

Table S1. TRN Reporting Guidelines

ITEM	DESCRIPTION
**Sample Type, Storage, Extraction, and Integrity**	
Sample type	Whole blood was collected in EDTA tubes. Approximately 200uL of whole blood from EDTA tubes were spotted on Whatman protein saver cards for dried blood spot (DBS) samples. Buffy coat cells were isolated using centrifugation to separate plasma followed by treatment with 0.5x red blood cell lysis buffer (Invitrogen). PBMCs were isolated using Ficoll gradient separation. Saliva was collected in Oragene kits (OGR-500, NA Genotek). Buccal cells were collected using sanitary swabs (Isohelix SK1).
Sample storage conditions	Buffy coat cells and PBMCs were stored at -80°C in a solution buffer comprised of phosphate buffered saline pH 7.2+EDTA (2mMol)+bovine serum albumin (0.5%) prior to extraction. DBS cards stored in sealed Ziploc bags with desiccant packets at room temperature. Buccal swabs were placed in sealed Ziploc bags and stored at -80°C. Saliva samples were aliquoted into 4 cryovials and stored at -80°C in Oragene stabilizing solution. The average duration between sample collection and DNA extraction was 1.83 years.
DNA extraction method	DNA was extracted from PBMCs, buffy coat cells, buccal swabs, saliva, and DBS cards using Gentra Puregene salting-out method following factory guidelines (Qiagen).
DNA storage conditions, including freeze-thaw cycles	DNA was stored at -80°C in Qiagen DNA Hydration Solution. On average there were three freeze thaws for DNA samples between extraction and the qPCR assay. The first thaw was done to determine DNA concentration using Quant-iT PicoGreen reagent (Qiagen). PicoGreen assays occurred in one batch with an average duration of 10.14 months between DNA extraction and PicoGreen assays.<br><br>A second freeze thaw was needed to perform a dilution for the qPCR assay. The final thaw occurred when the sample was assayed. Samples needing to be reassessed on qPCR assays (n=37; 4.71%) were thawed one additional time. DNA samples were stored for an average of 7.54 months between the PicoGreen assay and qPCR assay.
Method of documenting DNA quality and integrity	DNA was quantified for all tissue type samples using Quant-iT PicoGreen Reagent (mean=59.68 ng/uL). DNA purity was assessed using 260/230 and 260/280 ratios for all tissue types ($mean_{260/230}$=1.17; $mean_{260/280}$=1.82). An additional subset of samples (n=391, 49.8%) were evaluated using the DNA Integrity Number generated by the Agilent 2200 TapeStation with $mean_{DIN}$=7.58, indicating intact, minimally degraded DNA. No exclusionary criteria was imposed prior to qPCR assays.
Percentage of samples specifically tested for DNA quality and integrity	All samples were subjected to quality control via evaluation of 260/280 and 260/230 ratios. A subset of samples (391/785=49.8%) were subjected to quality assessment via TapeStation.
**qPCR Assay**	

<table>
<tr><th>ITEM</th><th colspan="2">DESCRIPTION</th></tr>
<tr><td>Method (qPCR, MMqPCR, aTL, etc.)</td><td colspan="2">qPCR assays to calculate absolute telomere length (aTL) were structured such that each assay comprised two qPCR runs, one run quantifying telomere content in kilobases (T) and a second run quantifying genome copy number (S) using the single copy gene IFNB1. The two runs (T & S) were always performed on the same day using the same DNA aliquot which was stored at 4°C between runs (~2.5 hours). Each run hosted triplicate reactions of 22 samples, 6 standards, 5 positive controls, and 1 no template control on 100 well disks.

A total of 42 qPCR assays were performed across a period of 52 days from 5/17/2022 to 7/8/2022 for analysis of all samples.</td></tr>
<tr><td>PCR machine type</td><td colspan="2">Qiagen Rotor-Gene Q using 100 well disks</td></tr>
<tr><td>Source of master mix and reagents, and final reaction volume</td><td colspan="2">The final reaction mix for the telomeric and IFNB1 reactions contains 1x QuantiTect SYBR Green Master Mix (Qiagen), 0.2U Uracil Glycosylase (Thermo Fisher Scientific), 0.1 uM forward-reverse primer pair, and 6 ng DNA in a 20 uL reaction.

Primers are purchased from IDT in as pre-mixed pairs (RxnReady Primer Pool) with HPLC purification and 10uM concentration in IDTE Buffer pH 8.0.</td></tr>
<tr><td>Telomere primer sequences and concentration</td><td colspan="2">Forward Primer: 5'-CGG TTT GTT TGG GTT TGG GTT TGG GTT TGG GTT TGG GTT-3′

Reverse Primer: 5'-GGC TTG CCT TAC CCT TAC CCT TAC CCT TAC CCT TAC CCT-3′</td></tr>
<tr><td>Single copy gene name, primer sequences, and concentration</td><td colspan="2">IFNB1 Forward Primer: 5’-TGG CAC AAC AGG TAG TAG GCG ACA C-3’

IFNB1 Reverse Primer: 5’-GCA CAA CAG GAG AGC AAT TTG GAG GA-3’</td></tr>
<tr><td rowspan="3">Full PCR program description including temperature, times, and cycle numbers</td><td colspan="2">50°C – 2min
95°C – 15min</td></tr>
<tr><td>95°C – 15s
55°C for 1 min</td><td>40 cycles</td></tr>
<tr><td colspan="2">Melt 60°C to 99°C rising 1°C per step with 5 sec per step</td></tr>
<tr><td>PCR efficiency of single copy gene and telomere primers</td><td colspan="2">Telo: R^2 = 0.9986; Efficiency=2.03
IFNB1: R^2 = 0.9987; Efficiency=1.99</td></tr>
</table>

ITEM	DESCRIPTION
Source and concentration of control samples and standard curve	5 positive controls were randomly selected across tissue type samples to control for variation across T and S runs. Standards consisted of double-stranded oligomers purchased from IDT as lyophilized pellet with PAGE purification.<br><br>Standard curves for T runs consisted of 84 bp double-stranded oligomer comprised of 16 copies of canonical telomere repeat. Telomere Standard A had concentration 0.10 ng/uL, which equates to 5.86e+08 kb telomeric DNA when 46uL is used in the qPCR assay. A series of 1/10 serial dilutions were performed to generate a total of 6 standards for each T run comprising a range of 5.86e+08 to 5.86e+5.86e+03 kb telomeric DNA.<br><br>Standard curves for S runs consisted of 83 bp double-stranded oligomer corresponding to the region of IFNB1 genomic DNA flanked by IFNB1 primers. IFNB1 Standard 1 had concentration 0.00033 ng/uL, which equates to 1.18e+07 diploid genomes when 6uL is used in the qPCR assay. A series of 1/10 serial dilutions were performed to generate a total of 6 standards for each S run comprising a range of 1.18e+07 to 1.18e+02 diploid genome copies.
Telomere Standard Oligomer Sequences	Sense: 5’-CCC TAA CCC TAA CCC TAA CCC TAA CCC TAA CCC TAA CCC TAA CCC TAA CCC TAA CCC TAA CCC TAA CCC TAA CCC TAA CCC TAA-3’<br><br>Anti-sense: 5’-TTA GGG TTA GGG TTA GGG TTA GGG TTA GGG TTA GGG TTA GGG TTA GGG TTA GGG TTA GGG TTA GGG TTA GGG TTA GGG TTA GGG-3’
IFNB1 Standard Oligomer Sequences	Sense: 5-GCA CAA CAG GAG AGC AAT TTG GAG GAG ACA CTT GTT GGT CAT GTT GAC AAC ACG AAC AGT GTC GCC TAC TAC CTG TTG TGC CA-3’<br><br>Anti-sense: 5’-TGG CAC AAC AGG TAG TAG GCG ACA CTG TTC GTG TTG TCA ACA TGA CCA ACA AGT GTC TCC TCC AAA TTG CTC TCC TGT TGT GC-3’
**Data Analysis**	
Mean and standard deviation or median range of telomere lengths	*All Tissues:* 10.55 kb (4.51)<br>*PBMC:* 10.31 kb (3.07)<br>*Buffy Coat:* 13.08 kb (3.20)<br>*DBS:* 12.60 kb (3.80)<br>*Buccal:* 10.44 kb (3.75)<br>*Saliva:* 6.82 kb (4.76)
Number of sample replicates	Each sample was assessed for T and S on a single run with three replicates within the run. If the sample did not pass quality control criteria described below it was run a second time.
Level of independence of replicates	Replicates were drawn from the same DNA aliquot (i.e., the same tube).

ITEM	DESCRIPTION
Analytic method, considering replicate measurements, to determine final length	Estimates of kb telomeric DNA and genome copy number were calculated automatically based on the alignment of each sample with the standard curve. When applicable, estimates for the no template control were subtracted from estimates of the analytical samples prior to calculating aTL values. The average kb telomeric DNA estimates and genome copy number estimates across triplicate measurements were used to calculate aTL values.<br>$$aTL = \frac{Estimated\ kb\ Telomeric\ DNA}{Estimated\ Genome\ Copy\ Number \times 92}$$<br>The average kb telomeric DNA estimates and genome copy number estimates across replicates were used to calculate aTL values.
Method of accounting for variation between replicates	When the coefficient of variation across triplicate estimates of telomere content or genome copy number was greater than 15%, replicate estimates were evaluated based upon their deviation from mean across triplicates. If one replicate deviated from the mean by more than 15% it was considered an outlier and the mean was recalculated using two replicates. Excepting samples that were rerun or failed, an average of 4.8 T replicates and 2.3 S replicates were dropped per run (*in this case aTL values were calculated using the average across duplicate measures*). In this manner the average intra-run CV across replicate kb telomeric DNA estimates and genome copy number estimates was 5.71% and 4.69% respectively.<br><br>In the case where coefficient of variation across replicates was still greater than 15% after removal of a single outlier, or was greater than 15% without a clear outlier defined by the criteria above, the sample was reassessed for both telomere content and genome copy number, and subjected to the same quality control evaluation. A total of 47 samples (4.71%) were rerun a second time.<br><br>The specific breakdown of intra-run CV, replicates dropped, and samples rerun by tissue and cohort is provided below. Values are presented as tissue/cohort averages with standard error in parentheses.

	Buccal		Saliva		DBS		Buffy Coat	PBMC
Variable	Child	Adult	Child	Adult	Child	Adult	Child	Adult
CV_(T)	6.55 (.32)	5.30 (.39)	5.32 (.29)	5.32 (.35)	5.75 (.32)	5.01 (.32)	6.07 (.34)	5.80 (.37)
CV_(S)	4.81 (.24)	5.25 (.39)	5.18 (.29)	4.97 (.43)	4.14 (.20)	4.48 (.31)	4.14 (.23)	4.48 (.31)
Replicates Dropped (T)	24	22	22	16	34	19	29	14
Replicates Dropped (S)	14	13	21	11	8	7	7	5
Samples Rerun	10	6	8	3	10	1	5	5

ITEM	DESCRIPTION
Method of accounting for well position effects within plates	The unique rotary design of the Rotor Gene Q is optimized to minimize well position effects. As such no accounting for well position effects was performed.
Method of accounting for between plate effects	To control for inter-assay variability, the telomeric content and genome copy number were assessed for five control samples on each T run and each S run. For each run, the estimated telomeric content and genome copy number were divided by the average estimated telomeric content and genome copy number for all runs to get a normalizing factor for that sample on a given run. This was done for all controls to get an average normalizing factor for that run. Estimates for analytical samples were then divided by the normalization factor for a given run. The average inter-assay CV for aTL estimates of 5 control samples across all 42 assays was 8.95%.
% of samples repeated and % of samples failing QC and excluding from further analyses	47/785 =5.99% of samples repeated<br>20/785 = 2.55% of samples failed QC and excluded from analyses.<br>All samples that failed QC were from saliva (14 Child; 6 Adult)
Acceptable range of PCR efficiency for single copy gene and telomere primers	1.90 – 2.10 (5*% variation*)
ICCs of samples/study groups to address variability	A pseudo-random selection of samples (n=44; 6%) were reassessed on two assays for the explicit purposes of calculating the ICC. The 44 samples were balanced such that they included 11 of each sample type (i.e,. DBS, buccal, saliva, and PBMC+buffy coat). These assays were subject to the same control for within and between plate variation as described above. ICCs were calculated at the level of aTL values using the *rpt* function in R using a Gaussian data distribution. The ICC across 44 samples rerun for reproducibility was 0.772. When a 'Tissue' factor was added to the model the ICC decreased to 0.728.<br><br>An additional selection of samples (n=44; 6%) were re-extracted and reassessed for the purposes of calculating an ICC of TL measurements across repeated extractions as recommended by the Telomere Research Network. Buccal samples were not available for re-extraction so the 44 samples were selected such that they included 11 each of PBMCs, buffy coat, DBS, and saliva samples. The ICC for these re-extracted samples was 0.826, which decreased to 0.784 when a 'Tissue' factor was added to the model.<br><br>The overall ICC of all 88 samples reassessed a second time was 0.776, which decreased to 0.725 when a 'Tissue' factor was added to the model.

ITEM	DESCRIPTION
T/S ratio transformed to a z-score prior before comparison across methods/studies	N/A. No comparison across studies was conducted.
How samples nested within families were accounted for	Samples from the same individual were run on the same plate except in cases when a single tissue from a given individual needed to be rerun due to high intra-assay CV.
