# Supplementary material for: Cross-tissue comparison of telomere length and quality metrics of DNA among individuals aged 8 to 70 years
Source: PLoS One. 2024 Feb 22;19(2):e0290918. doi: 10.1371/journal.pone.0290918 (PMC10883573; doi:10.1371/journal.pone.0290918)
Supplement: S2 Table — Outliers are defined as values outside the range of (Q1-1.5IQR) to (Q3+1.5IQR) for each cohort-tissue subset of data points, where Q1 and Q3 are lower and upper quartiles respectively, and IQR is the interquartile ratio. Outlier values were winsorized to the boundary values of this range. 295/5891 (5.0%) datapoints were winsorized across the study. (PDF) [file pone.0290918.s002.pdf]

			DNA Integrity				DNA Quality		DNA Concentration (ng/µL)			
	Tissue	aTL	DIN	% Unfrag (>3000 bp)	% High Frag (250–3000 bp)	% Sev Frag (<250 bp)	A260/280	A260/230	Nanodrop	Picogreen	TapeStation	Total
Child	Buccal	2	1	1	1	1	4	2	10	2		24
	DBS	5	1				10	3	1	2		22
	Saliva	1	2	1	1	1	7	4	12	12	5	46
	Buffy			1	2	3	12	2	5	12	1	38
	Total	8	4	3	4	5	33	11	28	28	6	130
Adult	Buccal	1	12	1	4	5	2	2	2		4	33
	DBS	1		2	3	2	9		3	4	5	29
	Saliva	2	4	5	5	5	2	5	6	10	5	49
	PBMC	1	8	7	10	9	3		9	5	2	54
	Total	5	24	15	22	21	16	7	20	19	16	165
											Total winsorized values:	295
