# Supplementary material for: Cross-tissue comparison of telomere length and quality metrics of DNA among individuals aged 8 to 70 years
Source: PLoS One. 2024 Feb 22;19(2):e0290918. doi: 10.1371/journal.pone.0290918 (PMC10883573; doi:10.1371/journal.pone.0290918)
Supplement: S3 Table — (PDF) [file pone.0290918.s003.pdf]

		aTL			DIN			% Unfragmented			% High Fragmented			% Severe Fragmented		
Predictor	Level	β	SE	p	β	SE	p	β	SE	p	β	SE	p	β	SE	p
(Intercept)		13.58	0.45	<0.001*	5.44	0.18	<0.001*	50.94	2.39	<0.001*	25.88	1.68	<0.001*	7.42	0.68	<0.001*
Age		-0.13	0.01	<0.001*	0.01	0.00	0.01	0.14	0.06	0.02	-0.09	0.04	0.03	-0.03	0.02	0.10
Sex	Female	REFERENCE			REFERENCE			REFERENCE			REFERENCE			REFERENCE		
	Male	-0.57	0.34	0.09	-0.11	0.09	0.22	-1.62	1.20	0.18	1.12	0.87	0.20	0.32	0.34	0.36
Tissue	Buccal	REFERENCE			REFERENCE			REFERENCE			REFERENCE			REFERENCE		
	Saliva	-6.34	0.47	<0.001*	2.14	0.24	<0.001*	12.01	3.07	<0.001*	-4.53	2.12	0.03	0.82	0.86	0.34
	DBS	2.13	0.46	<0.001*	2.39	0.24	<0.001*	28.26	3.08	<0.001*	-16.39	2.13	<0.001*	-3.47	0.87	<0.001*
	Buffy	0.05	2.19	0.98	4.22	1.23	<0.001*	50.47	15.89	0.002*	-27.12	11.08	0.02	-6.88	4.48	0.13
	PBMC	2.10	1.02	0.04	3.73	0.32	<0.001*	39.59	4.04	<0.001*	-25.72	2.81	<0.001*	-7.36	1.14	<0.001*
Race	White	REFERENCE			REFERENCE			REFERENCE			REFERENCE			REFERENCE		
	Black	1.15	0.65	0.08	0.01	0.23	0.97	-2.19	3.10	0.48	1.90	2.24	0.40	0.53	0.88	0.55
	Other	0.12	0.47	0.80	-0.42	0.14	0.01	-3.94	1.90	0.04	2.20	1.38	0.11	0.83	0.54	0.13
Age x Tissue	Saliva	0.11	0.02	<0.001*	0.00	0.01	0.88	0.12	0.08	0.11	-0.07	0.05	0.18	-0.03	0.02	0.24
	DBS	0.00	0.02	0.98	0.00	0.01	0.86	-0.12	0.08	0.12	0.15	0.05	0.01	0.06	0.02	0.01
	Buffy	0.08	0.18	0.67	-0.10	0.10	0.33	-0.82	1.30	0.53	0.24	0.91	0.80	0.01	0.37	0.98
	PBMC	0.01	0.02	0.73	-0.01	0.01	0.07	-0.09	0.09	0.33	0.09	0.07	0.16	0.03	0.03	0.25

		A260/280			A260/230			Nanodrop conc.			Picogreen conc.			TapeStation conc.		
Predictor	Level	β	SE	p	β	SE	p	β	SE	p	β	SE	p	β	SE	p
(Intercept)		1.88	0.01	<0.001*	1.14	0.05	<0.001*	167.16	20.84	<0.001*	47.18	7.39	<0.001*	56.82	13.95	<0.001*
Age		0.00	0.00	<0.001*	-0.01	0.00	<0.001*	0.12	0.66	0.86	0.14	0.23	0.54	-0.05	0.34	0.89
Sex	Female	REFERENCE			REFERENCE			REFERENCE			REFERENCE			REFERENCE		
	Male	-0.01	0.01	0.11	-0.04	0.03	0.25	-2.68	11.70	0.82	-1.50	4.15	0.72	-8.92	6.71	0.19
Tissue	Buccal	REFERENCE			REFERENCE			REFERENCE			REFERENCE			REFERENCE		
	Saliva	-0.04	0.02	0.003*	0.05	0.06	0.45	-120.14	27.36	<0.001*	-44.59	9.71	<0.001*	-46.01	18.34	0.01
	DBS	-0.08	0.02	<0.001*	0.17	0.06	0.00	-139.32	27.44	<0.001*	-36.73	9.74	<0.001*	-39.45	18.42	0.03
	Buffy	-0.01	0.07	0.85	-0.10	0.28	0.73	-81.38	120.80	0.50	20.07	42.86	0.64	-138.91	93.90	0.14
	PBMC	-0.01	0.03	0.82	0.40	0.13	0.002*	213.48	56.92	<0.001*	111.04	20.20	<0.001*	132.73	24.05	<0.001*
Race	White	REFERENCE			REFERENCE			REFERENCE			REFERENCE			REFERENCE		
	Black	0.01	0.01	0.36	-0.04	0.06	0.50	20.73	22.33	0.35	4.48	7.92	0.57	2.94	17.36	0.87
	Other	-0.01	0.01	0.43	-0.03	0.05	0.57	-0.51	16.33	0.98	1.09	5.79	0.85	-6.52	10.66	0.54
Age x Tissue	Saliva	0.00	0.00	<0.001*	0.00	0.00	0.46	1.30	0.93	0.16	0.07	0.33	0.83	0.21	0.46	0.65
	DBS	0.00	0.00	<0.001*	0.00	0.00	0.06	-0.07	0.93	0.94	-0.15	0.33	0.64	-0.01	0.46	0.98
	Buffy	0.00	0.01	0.94	0.04	0.02	0.12	24.56	9.92	0.01	6.78	3.52	0.06	22.99	7.68	0.003*
	PBMC	0.00	0.00	0.05	0.00	0.00	0.20	-2.10	1.36	0.12	-0.54	0.48	0.27	-0.65	0.56	0.24
