# Supplementary material for: Cross-tissue comparison of telomere length and quality metrics of DNA among individuals aged 8 to 70 years
Source: PLoS One. 2024 Feb 22;19(2):e0290918. doi: 10.1371/journal.pone.0290918 (PMC10883573; doi:10.1371/journal.pone.0290918)
Supplement: S4 Table — Asterisks indicate significant p-values after adjusting for multiple comparisons using the Benjamini-Hochberg method and controlling false discovery rate (FDR) at < 0.01. (PDF) [file pone.0290918.s004.pdf]

DV	Contrast	Age	Estimate	SE	df	t ratio	p-value	p Adj	Sig p
aTL	Buccal - Saliva	23.85	3.80	0.28	557.00	13.56	0.00	0.00	*
	Buccal - DBS	23.85	-2.14	0.27	557.00	-7.84	0.00	0.00	*
	Buccal - Buffy	23.85	-1.86	2.17	557.00	-0.86	0.39	0.52	
	Buccal - PBMC	23.85	-2.29	0.55	557.00	-4.17	0.00	0.00	*
	Saliva - DBS	23.85	-5.94	0.28	557.00	-21.14	0.00	0.00	*
	Saliva - Buffy	23.85	-5.66	2.17	557.00	-2.61	0.01	0.01	
	Saliva - PBMC	23.85	-6.09	0.55	557.00	-11.00	0.00	0.00	*
	DBS - Buffy	23.85	0.27	2.17	557.00	0.13	0.90	0.93	
	DBS - PBMC	23.85	-0.16	0.55	557.00	-0.28	0.78	0.86	
	Buffy - PBMC	23.85	-0.43	2.22	557.00	-0.19	0.85	0.91	
DIN	Buccal - Saliva	35.27	-2.17	0.11	280.00	-19.06	0.00	0.00	*
	Buccal - DBS	35.27	-2.35	0.11	280.00	-20.74	0.00	0.00	*
	Buccal - Buffy	35.27	-0.74	2.35	280.00	-0.31	0.75	0.84	
	Buccal - PBMC	35.27	-3.26	0.13	280.00	-25.26	0.00	0.00	*
	Saliva - DBS	35.27	-0.18	0.12	280.00	-1.55	0.12	0.17	
	Saliva - Buffy	35.27	1.43	2.35	280.00	0.61	0.54	0.66	
	Saliva - PBMC	35.27	-1.09	0.13	280.00	-8.33	0.00	0.00	*
	DBS - Buffy	35.27	1.61	2.35	280.00	0.69	0.49	0.62	
	DBS - PBMC	35.27	-0.91	0.13	280.00	-6.99	0.00	0.00	*
	Buffy - PBMC	35.27	-2.52	2.35	280.00	-1.07	0.28	0.40	
% Unfragmented DNA (>3000 bp)	Buccal - Saliva	35.21	-16.32	1.44	288.00	-11.34	0.00	0.00	*
	Buccal - DBS	35.21	-24.07	1.44	288.00	-16.68	0.00	0.00	*
	Buccal - Buffy	35.21	-21.71	30.23	288.00	-0.72	0.47	0.60	
	Buccal - PBMC	35.21	-36.40	1.64	288.00	-22.18	0.00	0.00	*
	Saliva - DBS	35.21	-7.75	1.44	288.00	-5.37	0.00	0.00	*
	Saliva - Buffy	35.21	-5.40	30.23	288.00	-0.18	0.86	0.91	
	Saliva - PBMC	35.21	-20.08	1.64	288.00	-12.24	0.00	0.00	*
	DBS - Buffy	35.21	2.35	30.23	288.00	0.08	0.94	0.96	
	DBS - PBMC	35.21	-12.33	1.64	288.00	-7.50	0.00	0.00	*
	Buffy - PBMC	35.21	-14.69	30.24	288.00	-0.49	0.63	0.74	
% Highly Fragmented DNA (250 – 3000 bp)	Buccal - Saliva	35.21	7.04	1.00	288.00	7.06	0.00	0.00	*
	Buccal - DBS	35.21	11.24	1.00	288.00	11.26	0.00	0.00	*
	Buccal - Buffy	35.21	18.82	21.09	288.00	0.89	0.37	0.50	
	Buccal - PBMC	35.21	22.51	1.14	288.00	19.78	0.00	0.00	*
	Saliva - DBS	35.21	4.21	1.00	288.00	4.22	0.00	0.00	*
	Saliva - Buffy	35.21	11.78	21.09	288.00	0.56	0.58	0.69	
	Saliva - PBMC	35.21	15.47	1.14	288.00	13.60	0.00	0.00	*
	DBS - Buffy	35.21	7.57	21.09	288.00	0.36	0.72	0.82	
	DBS - PBMC	35.21	11.26	1.14	288.00	9.87	0.00	0.00	*
	Buffy - PBMC	35.21	3.69	21.10	288.00	0.17	0.86	0.91	
% Severely Fragmented	Buccal - Saliva	35.21	0.08	0.40	288.00	0.20	0.84	0.91	
	Buccal - DBS	35.21	1.36	0.41	288.00	3.34	0.00	0.00	*

DNA (<250 bp)	Buccal - Buffy	35.21	6.49	8.52	288.00	0.76	0.45	0.58	
	Buccal - PBMC	35.21	6.29	0.46	288.00	13.63	0.00	0.00	*
	Saliva - DBS	35.21	1.28	0.41	288.00	3.14	0.00	0.00	*
	Saliva - Buffy	35.21	6.41	8.52	288.00	0.75	0.45	0.58	
	Saliva - PBMC	35.21	6.21	0.46	288.00	13.46	0.00	0.00	*
	DBS - Buffy	35.21	5.13	8.52	288.00	0.60	0.55	0.66	
	DBS - PBMC	35.21	4.94	0.46	288.00	10.67	0.00	0.00	*
	Buffy - PBMC	35.21	-0.20	8.52	288.00	-0.02	0.98	0.99	
A260/280	Buccal - Saliva	23.72	-0.02	0.01	577.00	-2.21	0.03	0.04	
	Buccal - DBS	23.72	0.12	0.01	577.00	13.56	0.00	0.00	*
	Buccal - Buffy	23.72	0.02	0.06	577.00	0.35	0.73	0.82	
	Buccal - PBMC	23.72	-0.03	0.02	577.00	-1.61	0.11	0.16	
	Saliva - DBS	23.72	0.14	0.01	577.00	15.79	0.00	0.00	*
	Saliva - Buffy	23.72	0.04	0.06	577.00	0.66	0.51	0.63	
	Saliva - PBMC	23.72	-0.01	0.02	577.00	-0.45	0.66	0.76	
	DBS - Buffy	23.72	-0.10	0.06	577.00	-1.54	0.12	0.17	
	DBS - PBMC	23.72	-0.15	0.02	577.00	-8.79	0.00	0.00	*
	Buffy - PBMC	23.72	-0.05	0.07	577.00	-0.75	0.45	0.58	
A260/230	Buccal - Saliva	23.72	-0.08	0.04	577.00	-2.26	0.02	0.04	
	Buccal - DBS	23.72	-0.08	0.04	577.00	-2.24	0.03	0.04	
	Buccal - Buffy	23.72	-0.74	0.27	577.00	-2.74	0.01	0.01	
	Buccal - PBMC	23.72	-0.49	0.07	577.00	-6.96	0.00	0.00	*
	Saliva - DBS	23.72	0.00	0.04	577.00	0.01	0.99	0.99	
	Saliva - Buffy	23.72	-0.66	0.27	577.00	-2.44	0.01	0.02	
	Saliva - PBMC	23.72	-0.41	0.07	577.00	-5.81	0.00	0.00	*
	DBS - Buffy	23.72	-0.66	0.27	577.00	-2.44	0.01	0.02	
	DBS - PBMC	23.72	-0.41	0.07	577.00	-5.81	0.00	0.00	*
	Buffy - PBMC	23.72	0.25	0.28	577.00	0.91	0.37	0.50	
Nanodrop DNA Concentration (ng/µL)	Buccal - Saliva	23.72	89.24	16.36	577.00	5.45	0.00	0.00	*
	Buccal - DBS	23.72	140.98	16.41	577.00	8.59	0.00	0.00	*
	Buccal - Buffy	23.72	-501.05	117.86	577.00	-4.25	0.00	0.00	*
	Buccal - PBMC	23.72	-163.80	31.01	577.00	-5.28	0.00	0.00	*
	Saliva - DBS	23.72	51.74	16.38	577.00	3.16	0.00	0.00	*
	Saliva - Buffy	23.72	-590.29	117.86	577.00	-5.01	0.00	0.00	*
	Saliva - PBMC	23.72	-253.04	31.00	577.00	-8.16	0.00	0.00	*
	DBS - Buffy	23.72	-642.03	117.86	577.00	-5.45	0.00	0.00	*
	DBS - PBMC	23.72	-304.78	31.02	577.00	-9.83	0.00	0.00	*
	Buffy - PBMC	23.72	337.25	120.76	577.00	2.79	0.01	0.01	*
PicoGreen DNA Concentration (ng/µL)	Buccal - Saliva	23.72	42.87	5.81	577.00	7.38	0.00	0.00	*
	Buccal - DBS	23.72	40.35	5.82	577.00	6.93	0.00	0.00	*
	Buccal - Buffy	23.72	-180.82	41.82	577.00	-4.32	0.00	0.00	*
	Buccal - PBMC	23.72	-98.30	11.00	577.00	-8.94	0.00	0.00	*
	Saliva - DBS	23.72	-2.52	5.81	577.00	-0.43	0.67	0.76	
	Saliva - Buffy	23.72	-223.69	41.82	577.00	-5.35	0.00	0.00	*

	Saliva - PBMC	23.72	-141.17	11.00	577.00	-12.84	0.00	0.00	*
	DBS - Buffy	23.72	-221.17	41.82	577.00	-5.29	0.00	0.00	*
	DBS - PBMC	23.72	-138.65	11.01	577.00	-12.60	0.00	0.00	*
	Buffy - PBMC	23.72	82.51	42.84	577.00	1.93	0.05	0.08	
TapeStation DNA Concentration (ng/μL)	Buccal - Saliva	35.21	38.58	8.61	288.00	4.48	0.00	0.00	*
	Buccal - DBS	35.21	39.91	8.63	288.00	4.62	0.00	0.00	*
	Buccal - Buffy	35.21	-670.54	178.56	288.00	-3.76	0.00	0.00	*
	Buccal - PBMC	35.21	-109.86	9.79	288.00	-11.23	0.00	0.00	*
	Saliva - DBS	35.21	1.33	8.63	288.00	0.15	0.88	0.91	
	Saliva - Buffy	35.21	-709.12	178.56	288.00	-3.97	0.00	0.00	*
	Saliva - PBMC	35.21	-148.45	9.79	288.00	-15.17	0.00	0.00	*
	DBS - Buffy	35.21	-710.45	178.56	288.00	-3.98	0.00	0.00	*
	DBS - PBMC	35.21	-149.78	9.81	288.00	-15.27	0.00	0.00	*
	Buffy - PBMC	35.21	560.67	178.63	288.00	3.14	0.00	0.00	*
