# Supplementary material for: Cross-tissue comparison of telomere length and quality metrics of DNA among individuals aged 8 to 70 years
Source: PLoS One. 2024 Feb 22;19(2):e0290918. doi: 10.1371/journal.pone.0290918 (PMC10883573; doi:10.1371/journal.pone.0290918)
Supplement: S5 Table — P-values were adjusted for multiple comparisons using the Benjamini-Hochberg method. Asterisks indicate significant p-values after controlling false discovery rate (FDR) at < 0.01. (PDF) [file pone.0290918.s005.pdf]

	aTL			DIN			% Unfrag			% High Frag			% Severe Frag		
Predictor	df	F	p	df	F	p	df	F	p	df	F	p	df	F	p
(Intercept)	1, 215	1640.997	<0.001*	1, 214	29693.222	<0.001*	1, 221	12168.468	<0.001*	1, 221	691.979	<0.001*	1, 221	564.379	<0.001*
Age	1, 71	14.954	<0.001*	1, 71	0.396	0.531	1, 71	4.633	0.035	1, 71	1.357	0.248	1, 71	0.767	0.384
Sex	1, 71	4.76	0.032	1, 71	0.155	0.695	1, 71	1.212	0.275	1, 71	1.627	0.206	1, 71	0.717	0.4
Tissue	3, 215	60.707	<0.001*	3, 214	271.078	<0.001*	3, 221	170.116	<0.001*	3, 221	125.951	<0.001*	3, 221	77.997	<0.001*
Race	2, 71	0.899	0.412	2, 71	3.341	0.041	2, 71	0.972	0.383	2, 71	0.698	0.501	2, 71	0.757	0.473
Age x Tissue	3, 215	6.365	<0.001*	3, 214	1.505	0.214	3, 221	3.868	0.01	3, 221	3.867	0.01	3, 221	3.561	0.015

	A260/280			A260/230			Nanodrop			PicoGreen			TapeStation		
Predictor	df	F	p	df	F	p	df	F	p	df	F	p	df	F	p
(Intercept)	1, 221	83898.23	<0.001*	1, 221	1592.102	<0.001*	1, 221	537.644	<0.001*	1, 221	404.432	<0.001*	1, 221	324.636	<0.001*
Age	1, 71	2.226	0.14	1, 71	0.526	0.471	1, 71	1.157	0.286	1, 71	0.4	0.529	1, 71	0.787	0.378
Sex	1, 71	0.665	0.417	1, 71	2.197	0.143	1, 71	0.851	0.359	1, 71	1.942	0.168	1, 71	0.98	0.326
Tissue	3, 221	84.966	<0.001*	3, 221	51.733	<0.001*	3, 221	73.159	<0.001*	3, 221	139.123	<0.001*	3, 221	132.606	<0.001*
Race	2, 71	0.259	0.773	2, 71	0.824	0.443	2, 71	3.289	0.043	2, 71	0.69	0.505	2, 71	3.247	0.045
Age x Tissue	3, 221	0.762	0.517	3, 221	1.383	0.249	3, 221	2.6	0.053	3, 221	0.563	0.64	3, 221	1.033	0.379
