# Supplementary material for: Cross-tissue comparison of telomere length and quality metrics of DNA among individuals aged 8 to 70 years
Source: PLoS One. 2024 Feb 22;19(2):e0290918. doi: 10.1371/journal.pone.0290918 (PMC10883573; doi:10.1371/journal.pone.0290918)
Supplement: S6 Table — (PDF) [file pone.0290918.s006.pdf]

Predictor	Level	aTL β	aTL SE	aTL p	DIN β	DIN SE	DIN p	% Unfrag β	% Unfrag SE	% Unfrag p	% High Frag β	% High Frag SE	% High Frag p	% Severe Frag β	% Severe Frag SE	% Severe Frag p
(Intercept)		9.8	0.92	<0.001*	5.67	0.25	<0.001*	52.17	3.61	<0.001*	22.91	2.56	<0.001*	6.26	1.03	<0.001*
Age		-0.05	0.02	0.02	0.01	0.01	0.22	0.11	0.08	0.15	-0.03	0.06	0.58	-0.01	0.02	0.82
Sex	Female	REFERENCE			REFERENCE			REFERENCE			REFERENCE			REFERENCE		
	Male	-0.81	0.43	0.06	-0.06	0.09	0.52	-1.76	1.40	0.21	1.50	1.02	0.15	0.44	0.41	0.28
Tissue	Buccal	REFERENCE			REFERENCE			REFERENCE			REFERENCE			REFERENCE		
	Saliva	-3.83	1.03	<0.001*	2.23	0.34	<0.001*	10.85	4.65	0.02	-1.37	3.24	0.67	2.39	1.32	0.07
	DBS	3.13	1.02	0.002*	3.02	0.34	<0.001*	32.39	4.70	<0.001*	-14.30	3.28	<0.001*	-2.29	1.33	0.09
	PBMC	4.35	1.01	<0.001*	3.52	0.34	<0.001*	38.66	4.65	<0.001*	-23.16	3.24	<0.001*	-6.32	1.32	<0.001*
Race	White	REFERENCE			REFERENCE			REFERENCE			REFERENCE			REFERENCE		
	Black	1.69	1.38	0.23	0.17	0.29	0.56	-1.29	4.39	0.77	2.21	3.19	0.49	0.94	1.28	0.46
	Other	-0.12	0.70	0.87	-0.37	0.15	0.02	-3.14	2.25	0.17	1.69	1.64	0.31	0.70	0.65	0.29
Age x Tissue	Saliva	0.06	0.02	0.01	-0.001	0.01	0.89	0.14	0.10	0.16	-0.13	0.07	0.07	-0.06	0.03	0.06
	DBS	-0.02	0.02	0.39	-0.01	0.01	0.07	-0.20	0.10	0.05	0.11	0.07	0.15	0.04	0.03	0.21
	PBMC	-0.04	0.02	0.11	-0.01	0.01	0.22	-0.07	0.10	0.48	0.04	0.07	0.57	0.01	0.03	0.74

Predictor	Level	A260/280			A260/230			Nanodrop			Picogreen			TapeStation		
		β	SE	p	β	SE	p	β	SE	p	B	SE	p	β	SE	p
(Intercept)		1.80	0.04	<0.001*	0.87	0.13	<0.001*	214.55	39.44	<0.001*	61.23	15.73	<0.001*	57.46	18.06	0.002*
Age		0.00	0.001	0.92	-0.002	0.003	0.53	-0.70	0.85	0.41	-0.08	0.34	0.81	-0.09	0.39	0.82
Sex	Female	REFERENCE			REFERENCE			REFERENCE			REFERENCE			REFERENCE		
	Male	-0.01	0.01	0.42	-0.08	0.05	0.12	-11.61	13.64	0.40	-7.27	5.58	0.20	-5.62	6.58	0.40
Tissue	Buccal	REFERENCE			REFERENCE			REFERENCE			REFERENCE			REFERENCE		
	Saliva	0.14	0.05	0.01	-0.11	0.16	0.51	-158.16	53.89	0.004	-56.85	21.26	0.01	-54.65	24.08	0.02
	DBS	-0.10	0.05	0.06	0.12	0.16	0.48	-180.55	54.48	0.001*	-46.32	21.49	0.03	-44.80	24.35	0.07
	PBMC	0.07	0.05	0.17	0.58	0.16	<0.001*	168.64	53.89	0.002*	98.94	21.26	<0.001*	127.15	24.08	<0.001*
Race	White	REFERENCE			REFERENCE			REFERENCE			REFERENCE			REFERENCE		
	Black	0.03	0.04	0.54	0.04	0.15	0.79	86.18	42.82	0.05	18.89	17.51	0.28	44.26	20.67	0.04
	Other	-0.01	0.02	0.77	-0.09	0.08	0.22	-30.26	22.00	0.17	-3.17	8.98	0.73	-12.29	10.60	0.25
Age x Tissue	Saliva	-0.001	0.001	0.41	0.01	0.004	0.21	2.06	1.20	0.09	0.32	0.47	0.50	0.38	0.54	0.47
	DBS	-0.001	0.001	0.20	-0.003	0.004	0.45	0.75	1.21	0.54	0.04	0.48	0.94	0.09	0.54	0.86
	PBMC	0.00	0.001	0.95	0.00	0.004	0.93	-1.21	1.20	0.32	-0.30	0.48	0.53	-0.54	0.54	0.32
