# Supplementary material for: Cross-tissue comparison of telomere length and quality metrics of DNA among individuals aged 8 to 70 years
Source: PLoS One. 2024 Feb 22;19(2):e0290918. doi: 10.1371/journal.pone.0290918 (PMC10883573; doi:10.1371/journal.pone.0290918)
Supplement: S7 Table — Asterisks indicate significant p-values after adjusting for multiple comparisons using the Benjamini-Hochberg method and controlling false discovery rate (FDR) at < 0.01. (PDF) [file pone.0290918.s007.pdf]

DV	Contrast	Age	Estimate	SE	df	t ratio	p-value	p Adj	Sig p
aTL	Buccal - Saliva	42.43	1.39	0.36	215.00	3.90	0.00	0.00	*
	Buccal - DBS	42.43	-2.31	0.35	215.00	-6.59	0.00	0.00	*
	Buccal - PBMC	42.43	-2.81	0.35	215.00	-8.06	0.00	0.00	*
	Saliva - DBS	42.43	-3.70	0.36	215.00	-10.31	0.00	0.00	*
	Saliva - PBMC	42.43	-4.20	0.36	215.00	-11.76	0.00	0.00	*
	DBS - PBMC	42.43	-0.50	0.35	215.00	-1.44	0.15	0.16	
DIN	Buccal - Saliva	42.42	-2.19	0.12	214.00	-18.63	0.00	0.00	*
	Buccal - DBS	42.42	-2.44	0.12	214.00	-20.83	0.00	0.00	*
	Buccal - PBMC	42.42	-3.13	0.12	214.00	-26.91	0.00	0.00	*
	Saliva - DBS	42.42	-0.25	0.12	214.00	-2.10	0.04	0.04	
	Saliva - PBMC	42.42	-0.94	0.12	214.00	-7.96	0.00	0.00	*
	DBS - PBMC	42.42	-0.69	0.12	214.00	-5.86	0.00	0.00	*
% Unfragmented DNA (>3000 bp)	Buccal - Saliva	42.26	-16.95	1.61	221.00	-10.52	0.00	0.00	*
	Buccal - DBS	42.26	-23.83	1.62	221.00	-14.74	0.00	0.00	*
	Buccal - PBMC	42.26	-35.56	1.61	221.00	-22.07	0.00	0.00	*
	Saliva - DBS	42.26	-6.88	1.62	221.00	-4.26	0.00	0.00	*
	Saliva - PBMC	42.26	-18.62	1.61	221.00	-11.56	0.00	0.00	*
	DBS - PBMC	42.26	-11.73	1.62	221.00	-7.26	0.00	0.00	*
% Highly Fragmented DNA (250 – 3000 bp)	Buccal - Saliva	42.26	7.00	1.12	221.00	6.22	0.00	0.00	*
	Buccal - DBS	42.26	9.84	1.13	221.00	8.72	0.00	0.00	*
	Buccal - PBMC	42.26	21.42	1.12	221.00	19.05	0.00	0.00	*
	Saliva - DBS	42.26	2.85	1.13	221.00	2.52	0.01	0.02	
	Saliva - PBMC	42.26	14.42	1.12	221.00	12.83	0.00	0.00	*
	DBS - PBMC	42.26	11.58	1.13	221.00	10.26	0.00	0.00	*
% Severely Fragmented DNA (<250 bp)	Buccal - Saliva	42.26	0.00	0.46	221.00	-0.01	0.99	0.99	
	Buccal - DBS	42.26	0.74	0.46	221.00	1.60	0.11	0.12	
	Buccal - PBMC	42.26	5.90	0.46	221.00	12.92	0.00	0.00	*
	Saliva - DBS	42.26	0.74	0.46	221.00	1.61	0.11	0.12	
	Saliva - PBMC	42.26	5.90	0.46	221.00	12.93	0.00	0.00	*
	DBS - PBMC	42.26	5.16	0.46	221.00	11.27	0.00	0.00	*
A260/280	Buccal - Saliva	42.26	-0.10	0.02	221.00	-5.63	0.00	0.00	*
	Buccal - DBS	42.26	0.16	0.02	221.00	9.04	0.00	0.00	*
	Buccal - PBMC	42.26	-0.06	0.02	221.00	-3.79	0.00	0.00	*
	Saliva - DBS	42.26	0.25	0.02	221.00	14.65	0.00	0.00	*
	Saliva - PBMC	42.26	0.03	0.02	221.00	1.83	0.07	0.08	
	DBS - PBMC	42.26	-0.22	0.02	221.00	-12.82	0.00	0.00	*
A260/230	Buccal - Saliva	42.26	-0.08	0.06	221.00	-1.49	0.14	0.15	
	Buccal - DBS	42.26	0.00	0.06	221.00	0.03	0.98	0.99	
	Buccal - PBMC	42.26	-0.59	0.06	221.00	-10.56	0.00	0.00	*
	Saliva - DBS	42.26	0.08	0.06	221.00	1.51	0.13	0.15	
	Saliva - PBMC	42.26	-0.51	0.06	221.00	-9.08	0.00	0.00	*
	DBS - PBMC	42.26	-0.59	0.06	221.00	-10.55	0.00	0.00	*
Nanodrop DNA Concentration (ng/µL)	Buccal - Saliva	42.26	71.32	18.69	221.00	3.82	0.00	0.00	*
	Buccal - DBS	42.26	149.08	18.75	221.00	7.95	0.00	0.00	*
	Buccal - PBMC	42.26	-117.60	18.69	221.00	-6.29	0.00	0.00	*
	Saliva - DBS	42.26	77.76	18.75	221.00	4.15	0.00	0.00	*

	Saliva - PBMC	42.26	-188.92	18.69	221.00	-10.11	0.00	0.00	*
	DBS - PBMC	42.26	-266.67	18.75	221.00	-14.22	0.00	0.00	*
PicoGreen DNA Concentration (ng/µL)	Buccal - Saliva	42.26	43.49	7.37	221.00	5.90	0.00	0.00	*
	Buccal - DBS	42.26	44.75	7.40	221.00	6.05	0.00	0.00	*
	Buccal - PBMC	42.26	-86.41	7.37	221.00	-11.72	0.00	0.00	*
	Saliva - DBS	42.26	1.26	7.40	221.00	0.17	0.86	0.89	
	Saliva - PBMC	42.26	-129.89	7.37	221.00	-17.62	0.00	0.00	*
	DBS - PBMC	42.26	-131.15	7.40	221.00	-17.73	0.00	0.00	*
TapeStation DNA Concentration (ng/µL)	Buccal - Saliva	42.26	38.41	8.35	221.00	4.60	0.00	0.00	*
	Buccal - DBS	42.26	40.81	8.38	221.00	4.87	0.00	0.00	*
	Buccal - PBMC	42.26	-104.43	8.35	221.00	-12.51	0.00	0.00	*
	Saliva - DBS	42.26	2.40	8.38	221.00	0.29	0.77	0.82	
	Saliva - PBMC	42.26	-142.85	8.35	221.00	-17.11	0.00	0.00	*
	DBS - PBMC	42.26	-145.25	8.38	221.00	-17.34	0.00	0.00	*
