# Supplementary material for: Cross-tissue comparison of telomere length and quality metrics of DNA among individuals aged 8 to 70 years
Source: PLoS One. 2024 Feb 22;19(2):e0290918. doi: 10.1371/journal.pone.0290918 (PMC10883573; doi:10.1371/journal.pone.0290918)
Supplement: S8 Table — (PDF) [file pone.0290918.s008.pdf]

Tissue	Parameter1	Parameter2	rho	95% CI Low	95% CI High	S	p	p.adj
**Buccal**	DIN	%Unfrag	0.96	0.94	0.97	6956.00	<0.001	<0.0001
**Buccal**	DIN	%High Frag	-0.85	-0.90	-0.79	309058.00	<0.001	<0.0001
**Buccal**	DIN	%Severe Frag	-0.64	-0.75	-0.51	273936.00	<0.001	<0.0001
**Buccal**	DIN	A260/A280	0.22	0.02	0.41	129506.00	0.03	0.039
**Buccal**	DIN	A260/A230	0.23	0.03	0.41	128838.00	0.02	0.035
**Buccal**	DIN	Conc(Nano)	-0.05	-0.25	0.15	174850.00	0.63	0.688
**Buccal**	DIN	Conc(Pico)	-0.02	-0.22	0.18	169690.00	0.86	0.882
**Buccal**	DIN	Conc(Tape)	-0.03	-0.23	0.18	170962.00	0.80	0.840
**Buccal**	%Unfrag	%High Frag	-0.93	-0.95	-0.89	321298.00	<0.001	<0.0001
**Buccal**	%Unfrag	%Severe Frag	-0.75	-0.83	-0.65	292002.00	<0.001	<0.0001
**Buccal**	%Unfrag	A260/A280	0.26	0.06	0.44	123858.00	0.01	0.016
**Buccal**	%Unfrag	A260/A230	0.28	0.08	0.46	119814.00	<0.001	0.008
**Buccal**	%Unfrag	Conc(Nano)	0.02	-0.18	0.22	163302.00	0.84	0.877
**Buccal**	%Unfrag	Conc(Pico)	0.05	-0.16	0.25	158734.00	0.64	0.693
**Buccal**	%Unfrag	Conc(Tape)	0.05	-0.15	0.25	157640.00	0.59	0.663
**Buccal**	%High Frag	%Severe Frag	0.92	0.88	0.94	14056.00	<0.001	<0.0001
**Buccal**	%High Frag	A260/A280	-0.29	-0.47	-0.10	215522.00	<0.001	0.005
**Buccal**	%High Frag	A260/A230	-0.40	-0.55	-0.21	232546.00	<0.001	<0.0001
**Buccal**	%High Frag	Conc(Nano)	-0.21	-0.39	0.00	200972.00	0.04	0.058
**Buccal**	%High Frag	Conc(Pico)	-0.15	-0.34	0.05	191784.00	0.13	0.182
**Buccal**	%High Frag	Conc(Tape)	-0.14	-0.33	0.07	189390.00	0.18	0.231
**Buccal**	%Severe Frag	A260/A280	-0.28	-0.46	-0.08	213120.00	<0.001	0.008
**Buccal**	%Severe Frag	A260/A230	-0.48	-0.62	-0.31	247046.00	<0.001	<0.0001
**Buccal**	%Severe Frag	Conc(Nano)	-0.38	-0.54	-0.20	230804.00	<0.001	<0.0001
**Buccal**	%Severe Frag	Conc(Pico)	-0.30	-0.47	-0.10	216244.00	<0.001	0.005
**Buccal**	%Severe Frag	Conc(Tape)	-0.25	-0.43	-0.05	208738.00	0.01	0.018
**Buccal**	A260/A280	A260/A230	0.56	0.45	0.65	557361.72	<0.001	<0.0001
**Buccal**	A260/A280	Conc(Nano)	-0.20	-0.34	-0.06	1506738.10	<0.001	0.008
**Buccal**	A260/A280	Conc(Pico)	-0.27	-0.40	-0.13	1595317.14	<0.001	<0.0001
**Buccal**	A260/A280	Conc(Tape)	-0.09	-0.28	0.12	181196.00	0.39	0.457
**Buccal**	A260/A230	Conc(Nano)	0.25	0.11	0.38	939270.00	<0.001	0.001
**Buccal**	A260/A230	Conc(Pico)	0.22	0.08	0.35	982214.00	<0.001	0.004
**Buccal**	A260/A230	Conc(Tape)	0.32	0.13	0.49	113076.00	<0.001	0.002
**Buccal**	Conc(Nano)	Conc(Pico)	0.94	0.92	0.96	72394.00	<0.001	<0.0001
**Buccal**	Conc(Nano)	Conc(Tape)	0.76	0.66	0.84	39676.00	<0.001	<0.0001
**Buccal**	Conc(Pico)	Conc(Tape)	0.82	0.74	0.88	30166.00	<0.001	<0.0001
**Saliva**	DIN	%Unfrag	0.66	0.52	0.76	48666.00	<0.001	<0.0001
**Saliva**	DIN	%High Frag	-0.60	-0.72	-0.44	228054.00	<0.001	<0.0001
**Saliva**	DIN	%Severe Frag	-0.50	-0.64	-0.33	214720.00	<0.001	<0.0001

**Saliva**	DIN	A260/A280	0.01	-0.20	0.21	142040.00	0.95	0.966
**Saliva**	DIN	A260/A230	0.13	-0.08	0.33	124234.00	0.21	0.267
**Saliva**	DIN	Conc(Nano)	0.55	0.39	0.68	64042.00	<0.001	<0.0001
**Saliva**	DIN	Conc(Pico)	0.45	0.27	0.60	78270.00	<0.001	<0.0001
**Saliva**	DIN	Conc(Tape)	0.38	0.19	0.54	88718.00	<0.001	<0.0001
**Saliva**	%Unfrag	%High Frag	-0.94	-0.96	-0.91	323598.00	<0.001	<0.0001
**Saliva**	%Unfrag	%Severe Frag	-0.89	-0.92	-0.84	314760.00	<0.001	<0.0001
**Saliva**	%Unfrag	A260/A280	-0.15	-0.34	0.06	191054.00	0.15	0.194
**Saliva**	%Unfrag	A260/A230	0.37	0.19	0.54	104298.00	<0.001	<0.0001
**Saliva**	%Unfrag	Conc(Nano)	0.65	0.52	0.76	57668.00	<0.001	<0.0001
**Saliva**	%Unfrag	Conc(Pico)	0.73	0.62	0.81	44752.00	<0.001	<0.0001
**Saliva**	%Unfrag	Conc(Tape)	0.82	0.73	0.87	30712.00	<0.001	<0.0001
**Saliva**	%High Frag	%Severe Frag	0.98	0.96	0.98	4124.00	<0.001	<0.0001
**Saliva**	%High Frag	A260/A280	0.23	0.03	0.42	127806.00	0.02	0.031
**Saliva**	%High Frag	A260/A230	-0.49	-0.63	-0.32	248034.00	<0.001	<0.0001
**Saliva**	%High Frag	Conc(Nano)	-0.73	-0.82	-0.62	288952.00	<0.001	<0.0001
**Saliva**	%High Frag	Conc(Pico)	-0.80	-0.86	-0.71	299148.00	<0.001	<0.0001
**Saliva**	%High Frag	Conc(Tape)	-0.90	-0.93	-0.85	316178.00	<0.001	<0.0001
**Saliva**	%Severe Frag	A260/A280	0.27	0.07	0.45	121370.00	0.01	0.010
**Saliva**	%Severe Frag	A260/A230	-0.52	-0.66	-0.36	253894.00	<0.001	<0.0001
**Saliva**	%Severe Frag	Conc(Nano)	-0.75	-0.83	-0.65	291766.00	<0.001	<0.0001
**Saliva**	%Severe Frag	Conc(Pico)	-0.81	-0.87	-0.73	302440.00	<0.001	<0.0001
**Saliva**	%Severe Frag	Conc(Tape)	-0.92	-0.95	-0.88	319948.00	<0.001	<0.0001
**Saliva**	A260/A280	A260/A230	-0.01	-0.15	0.13	1286658.00	0.89	0.907
**Saliva**	A260/A280	Conc(Nano)	-0.03	-0.17	0.12	1310762.00	0.69	0.738
**Saliva**	A260/A280	Conc(Pico)	-0.06	-0.20	0.09	1347876.00	0.42	0.490
**Saliva**	A260/A280	Conc(Tape)	-0.32	-0.49	-0.13	220330.00	<0.001	0.002
**Saliva**	A260/A230	Conc(Nano)	0.24	0.10	0.37	969216.00	<0.001	0.001
**Saliva**	A260/A230	Conc(Pico)	0.38	0.25	0.50	789568.00	<0.001	<0.0001
**Saliva**	A260/A230	Conc(Tape)	0.56	0.41	0.69	73014.00	<0.001	<0.0001
**Saliva**	Conc(Nano)	Conc(Pico)	0.82	0.77	0.86	230816.00	<0.001	<0.0001
**Saliva**	Conc(Nano)	Conc(Tape)	0.73	0.62	0.81	44990.00	<0.001	<0.0001
**Saliva**	Conc(Pico)	Conc(Tape)	0.85	0.78	0.90	25052.00	<0.001	<0.0001
**DBS**	DIN	%Unfrag	0.72	0.61	0.81	42116.00	<0.001	<0.0001
**DBS**	DIN	%High Frag	-0.58	-0.70	-0.42	239594.00	<0.001	<0.0001
**DBS**	DIN	%Severe Frag	-0.42	-0.58	-0.24	216116.00	<0.001	<0.0001
**DBS**	DIN	A260/A280	0.08	-0.13	0.28	139838.00	0.43	0.502
**DBS**	DIN	A260/A230	-0.11	-0.31	0.10	169142.00	0.27	0.341
**DBS**	DIN	Conc(Nano)	0.16	-0.05	0.35	127748.00	0.12	0.161
**DBS**	DIN	Conc(Pico)	0.42	0.23	0.57	88460.00	<0.001	<0.0001
**DBS**	DIN	Conc(Tape)	0.37	0.17	0.53	96530.00	<0.001	<0.0001

**DBS**	%Unfrag	%High Frag	-0.95	-0.97	-0.93	315326.00	<0.001	<0.0001
**DBS**	%Unfrag	%Severe Frag	-0.84	-0.89	-0.77	297634.00	<0.001	<0.0001
**DBS**	%Unfrag	A260/A280	-0.10	-0.30	0.11	177558.00	0.33	0.405
**DBS**	%Unfrag	A260/A230	-0.15	-0.34	0.05	186284.00	0.13	0.181
**DBS**	%Unfrag	Conc(Nano)	0.42	0.24	0.57	93956.00	<0.001	<0.0001
**DBS**	%Unfrag	Conc(Pico)	0.73	0.62	0.81	43158.00	<0.001	<0.0001
**DBS**	%Unfrag	Conc(Tape)	0.82	0.74	0.88	29492.00	<0.001	<0.0001
**DBS**	%High Frag	%Severe Frag	0.92	0.88	0.94	13414.00	<0.001	<0.0001
**DBS**	%High Frag	A260/A280	0.07	-0.14	0.27	150514.00	0.50	0.558
**DBS**	%High Frag	A260/A230	0.09	-0.11	0.29	146908.00	0.37	0.438
**DBS**	%High Frag	Conc(Nano)	-0.48	-0.62	-0.31	239102.00	<0.001	<0.0001
**DBS**	%High Frag	Conc(Pico)	-0.77	-0.84	-0.67	285950.00	<0.001	<0.0001
**DBS**	%High Frag	Conc(Tape)	-0.91	-0.94	-0.86	308074.00	<0.001	<0.0001
**DBS**	%Severe Frag	A260/A280	0.12	-0.09	0.31	143054.00	0.26	0.322
**DBS**	%Severe Frag	A260/A230	0.13	-0.07	0.33	140418.00	0.19	0.251
**DBS**	%Severe Frag	Conc(Nano)	-0.50	-0.63	-0.33	241854.00	<0.001	<0.0001
**DBS**	%Severe Frag	Conc(Pico)	-0.72	-0.80	-0.60	277776.00	<0.001	<0.0001
**DBS**	%Severe Frag	Conc(Tape)	-0.91	-0.94	-0.87	308696.00	<0.001	<0.0001
**DBS**	A260/A280	A260/A230	0.53	0.42	0.63	575576.73	<0.001	<0.0001
**DBS**	A260/A280	Conc(Nano)	0.15	0.01	0.29	1049212.92	0.04	0.053
**DBS**	A260/A280	Conc(Pico)	0.19	0.04	0.32	1004801.91	0.01	0.014
**DBS**	A260/A280	Conc(Tape)	-0.11	-0.30	0.10	178874.00	0.30	0.362
**DBS**	A260/A230	Conc(Nano)	-0.10	-0.25	0.04	1364874.00	0.15	0.194
**DBS**	A260/A230	Conc(Pico)	0.01	-0.13	0.16	1221172.00	0.87	0.889
**DBS**	A260/A230	Conc(Tape)	-0.07	-0.27	0.13	173560.00	0.47	0.533
**DBS**	Conc(Nano)	Conc(Pico)	0.68	0.60	0.75	390494.00	<0.001	<0.0001
**DBS**	Conc(Nano)	Conc(Tape)	0.55	0.39	0.68	72672.00	<0.001	<0.0001
**DBS**	Conc(Pico)	Conc(Tape)	0.81	0.72	0.87	31494.00	<0.001	<0.0001
**Buffy**	DIN	%Unfrag	0.48	0.07	0.75	1056.00	0.02	0.032
**Buffy**	DIN	%High Frag	-0.36	-0.68	0.07	2752.00	0.09	0.130
**Buffy**	DIN	%Severe Frag	-0.19	-0.57	0.25	2406.00	0.39	0.457
**Buffy**	DIN	A260/A280	-0.20	-0.58	0.24	2434.00	0.35	0.425
**Buffy**	DIN	A260/A230	0.34	-0.09	0.67	1326.00	0.11	0.149
**Buffy**	DIN	Conc(Nano)	0.04	-0.39	0.46	1942.00	0.85	0.882
**Buffy**	DIN	Conc(Pico)	0.25	-0.19	0.61	1518.00	0.25	0.319
**Buffy**	DIN	Conc(Tape)	0.16	-0.28	0.55	1692.00	0.45	0.521
**Buffy**	%Unfrag	%High Frag	-0.86	-0.94	-0.69	3770.00	<0.001	<0.0001
**Buffy**	%Unfrag	%Severe Frag	-0.71	-0.87	-0.41	3456.00	<0.001	<0.0001
**Buffy**	%Unfrag	A260/A280	-0.01	-0.43	0.42	2040.00	0.97	0.975
**Buffy**	%Unfrag	A260/A230	0.24	-0.20	0.60	1540.00	0.27	0.340
**Buffy**	%Unfrag	Conc(Nano)	0.01	-0.42	0.43	2010.00	0.98	0.975

**Buffy**	%Unfrag	Conc(Pico)	0.16	-0.28	0.55	1698.00	0.46	0.527
**Buffy**	%Unfrag	Conc(Tape)	0.60	0.24	0.82	810.00	<0.001	0.005
**Buffy**	%High Frag	%Severe Frag	0.90	0.77	0.96	204.00	<0.001	<0.0001
**Buffy**	%High Frag	A260/A280	-0.11	-0.51	0.33	2244.00	0.62	0.686
**Buffy**	%High Frag	A260/A230	-0.39	-0.70	0.04	2810.00	0.07	0.097
**Buffy**	%High Frag	Conc(Nano)	-0.05	-0.46	0.38	2120.00	0.83	0.868
**Buffy**	%High Frag	Conc(Pico)	-0.24	-0.60	0.21	2500.00	0.28	0.345
**Buffy**	%High Frag	Conc(Tape)	-0.75	-0.89	-0.49	3550.00	<0.001	<0.0001
**Buffy**	%Severe Frag	A260/A280	-0.06	-0.47	0.37	2154.00	0.77	0.816
**Buffy**	%Severe Frag	A260/A230	-0.44	-0.73	-0.02	2906.00	0.04	0.056
**Buffy**	%Severe Frag	Conc(Nano)	-0.21	-0.58	0.23	2450.00	0.34	0.405
**Buffy**	%Severe Frag	Conc(Pico)	-0.31	-0.65	0.13	2656.00	0.15	0.194
**Buffy**	%Severe Frag	Conc(Tape)	-0.85	-0.94	-0.68	3754.00	<0.001	<0.0001
**Buffy**	A260/A280	A260/A230	-0.44	-0.58	-0.28	415556.00	<0.001	<0.0001
**Buffy**	A260/A280	Conc(Nano)	-0.56	-0.67	-0.41	448082.00	<0.001	<0.0001
**Buffy**	A260/A280	Conc(Pico)	-0.51	-0.64	-0.36	436220.00	<0.001	<0.0001
**Buffy**	A260/A280	Conc(Tape)	-0.10	-0.51	0.33	2236.00	0.63	0.692
**Buffy**	A260/A230	Conc(Nano)	0.63	0.50	0.73	107966.00	<0.001	<0.0001
**Buffy**	A260/A230	Conc(Pico)	0.56	0.42	0.68	125406.00	<0.001	<0.0001
**Buffy**	A260/A230	Conc(Tape)	0.56	0.18	0.80	888.00	0.01	0.009
**Buffy**	Conc(Nano)	Conc(Pico)	0.75	0.66	0.82	71822.00	<0.001	<0.0001
**Buffy**	Conc(Nano)	Conc(Tape)	0.42	-0.01	0.71	1184.00	0.05	0.071
**Buffy**	Conc(Pico)	Conc(Tape)	0.37	-0.06	0.69	1266.00	0.08	0.112
**PBMC**	DIN	%Unfrag	0.31	0.08	0.50	50802.00	0.01	0.012
**PBMC**	DIN	%High Frag	-0.48	-0.64	-0.28	108208.00	<0.001	<0.0001
**PBMC**	DIN	%Severe Frag	-0.32	-0.51	-0.09	96534.00	<0.001	0.008
**PBMC**	DIN	A260/A280	0.06	-0.18	0.29	68900.00	0.62	0.686
**PBMC**	DIN	A260/A230	0.40	0.19	0.58	43856.00	<0.001	0.001
**PBMC**	DIN	Conc(Nano)	0.43	0.22	0.60	41834.00	<0.001	<0.0001
**PBMC**	DIN	Conc(Pico)	0.19	-0.05	0.40	59498.00	0.11	0.149
**PBMC**	DIN	Conc(Tape)	0.32	0.09	0.51	49948.00	0.01	0.009
**PBMC**	%Unfrag	%High Frag	-0.67	-0.78	-0.53	127342.00	<0.001	<0.0001
**PBMC**	%Unfrag	%Severe Frag	-0.57	-0.71	-0.39	119582.00	<0.001	<0.0001
**PBMC**	%Unfrag	A260/A280	0.09	-0.15	0.31	69374.00	0.45	0.515
**PBMC**	%Unfrag	A260/A230	0.24	0.01	0.44	58170.00	0.04	0.058
**PBMC**	%Unfrag	Conc(Nano)	0.04	-0.19	0.27	72988.00	0.73	0.773
**PBMC**	%Unfrag	Conc(Pico)	0.15	-0.08	0.37	64684.00	0.19	0.251
**PBMC**	%Unfrag	Conc(Tape)	0.41	0.19	0.58	45258.00	<0.001	0.001
**PBMC**	%High Frag	%Severe Frag	0.90	0.85	0.94	7326.00	<0.001	<0.0001
**PBMC**	%High Frag	A260/A280	0.05	-0.18	0.28	72062.00	0.65	0.699
**PBMC**	%High Frag	A260/A230	-0.39	-0.57	-0.18	105872.00	<0.001	0.001

**PBMC**	%High Frag	Conc(Nano)	-0.43	-0.60	-0.22	108454.00	<0.001	<0.0001
**PBMC**	%High Frag	Conc(Pico)	-0.43	-0.60	-0.22	108526.00	<0.001	<0.0001
**PBMC**	%High Frag	Conc(Tape)	-0.73	-0.82	-0.60	131526.00	<0.001	<0.0001
**PBMC**	%Severe Frag	A260/A280	0.18	-0.05	0.40	62068.00	0.11	0.151
**PBMC**	%Severe Frag	A260/A230	-0.46	-0.62	-0.26	111140.00	<0.001	<0.0001
**PBMC**	%Severe Frag	Conc(Nano)	-0.49	-0.65	-0.30	113590.00	<0.001	<0.0001
**PBMC**	%Severe Frag	Conc(Pico)	-0.55	-0.69	-0.37	117868.00	<0.001	<0.0001
**PBMC**	%Severe Frag	Conc(Tape)	-0.83	-0.89	-0.74	138890.00	<0.001	<0.0001
**PBMC**	A260/A280	A260/A230	-0.13	-0.35	0.10	86094.00	0.25	0.321
**PBMC**	A260/A280	Conc(Nano)	-0.33	-0.52	-0.11	101168.00	<0.001	0.006
**PBMC**	A260/A280	Conc(Pico)	-0.32	-0.51	-0.09	100186.00	<0.001	0.008
**PBMC**	A260/A280	Conc(Tape)	-0.26	-0.47	-0.04	96122.00	0.02	0.032
**PBMC**	A260/A230	Conc(Nano)	0.67	0.53	0.78	24754.00	<0.001	<0.0001
**PBMC**	A260/A230	Conc(Pico)	0.68	0.54	0.79	24212.00	<0.001	<0.0001
**PBMC**	A260/A230	Conc(Tape)	0.57	0.39	0.71	32934.00	<0.001	<0.0001
**PBMC**	Conc(Nano)	Conc(Pico)	0.79	0.68	0.86	16244.00	<0.001	<0.0001
**PBMC**	Conc(Nano)	Conc(Tape)	0.60	0.43	0.73	30628.00	<0.001	<0.0001
**PBMC**	Conc(Pico)	Conc(Tape)	0.64	0.48	0.76	27568.00	<0.001	<0.0001
