# Supplementary material for: Cross-tissue comparison of telomere length and quality metrics of DNA among individuals aged 8 to 70 years
Source: PLoS One. 2024 Feb 22;19(2):e0290918. doi: 10.1371/journal.pone.0290918 (PMC10883573; doi:10.1371/journal.pone.0290918)
Supplement: S9 Table — (PDF) [file pone.0290918.s009.pdf]

Tissue	Parameter1	Parameter2	rho	95% CI Low	95% CI High	S	p	p.adj
**Buccal**	DIN	aTL_win	-0.26	-0.44	-0.06	209818.00	0.009	0.025
**Buccal**	%Unfrag	aTL_win	-0.21	-0.39	-0.01	201336.00	0.038	0.081
**Buccal**	%High Frag	aTL_win	0.19	-0.01	0.38	134748.00	0.056	0.102
**Buccal**	%Severe Frag	aTL_win	0.09	-0.12	0.28	152364.00	0.396	0.482
**Buccal**	A260/A280	aTL_win	0.05	-0.09	0.19	1189497.97	0.468	0.540
**Buccal**	A260/A230	aTL_win	0.25	0.11	0.38	936474.00	<0.0001	0.001
**Buccal**	Conc(Nano)	aTL_win	0.30	0.16	0.42	883508.00	<0.0001	<0.0001
**Buccal**	Conc(Pico)	aTL_win	0.29	0.16	0.42	886546.00	<0.0001	<0.0001
**Buccal**	Conc(Tape)	aTL_win	0.24	0.04	0.42	127306.00	0.018	0.043
**Saliva**	DIN	aTL_win	0.20	-0.01	0.39	100650.00	0.059	0.103
**Saliva**	%Unfrag	aTL_win	0.56	0.40	0.69	59152.00	<0.0001	<0.0001
**Saliva**	%High Frag	aTL_win	-0.63	-0.74	-0.48	217844.00	<0.0001	<0.0001
**Saliva**	%Severe Frag	aTL_win	-0.66	-0.77	-0.52	222736.00	<0.0001	<0.0001
**Saliva**	A260/A280	aTL_win	-0.03	-0.18	0.13	948316.00	0.730	0.782
**Saliva**	A260/A230	aTL_win	0.18	0.03	0.32	756986.00	0.016	0.040
**Saliva**	Conc(Nano)	aTL_win	0.54	0.42	0.64	428194.00	<0.0001	<0.0001
**Saliva**	Conc(Pico)	aTL_win	0.59	0.48	0.68	380954.00	<0.0001	<0.0001
**Saliva**	Conc(Tape)	aTL_win	0.62	0.47	0.73	50918.00	<0.0001	<0.0001
**DBS**	DIN	aTL_win	-0.03	-0.23	0.18	156350.00	0.786	0.804
**DBS**	%Unfrag	aTL_win	0.15	-0.06	0.34	137746.00	0.143	0.208
**DBS**	%High Frag	aTL_win	-0.27	-0.45	-0.07	205574.00	0.007	0.019
**DBS**	%Severe Frag	aTL_win	-0.33	-0.50	-0.14	215196.00	0.001	0.003
**DBS**	A260/A280	aTL_win	0.05	-0.09	0.20	1170010.97	0.460	0.540
**DBS**	A260/A230	aTL_win	0.15	0.00	0.29	1053544.00	0.040	0.081
**DBS**	Conc(Nano)	aTL_win	0.02	-0.13	0.16	1211662.00	0.787	0.804
**DBS**	Conc(Pico)	aTL_win	0.04	-0.10	0.19	1181314.00	0.541	0.608
**DBS**	Conc(Tape)	aTL_win	0.37	0.18	0.53	102376.00	<0.0001	0.001
**Buffy**	DIN	aTL_win	-0.26	-0.62	0.18	2554.00	0.227	0.301
**Buffy**	%Unfrag	aTL_win	0.01	-0.42	0.43	2012.00	0.979	0.979
**Buffy**	%High Frag	aTL_win	-0.20	-0.58	0.24	2434.00	0.354	0.442
**Buffy**	%Severe Frag	aTL_win	-0.24	-0.60	0.21	2500.00	0.280	0.360
**Buffy**	A260/A280	aTL_win	-0.18	-0.36	0.00	341000.00	0.044	0.086
**Buffy**	A260/A230	aTL_win	0.36	0.18	0.51	185636.00	<0.0001	<0.0001
**Buffy**	Conc(Nano)	aTL_win	0.42	0.25	0.56	167880.00	<0.0001	<0.0001
**Buffy**	Conc(Pico)	aTL_win	0.30	0.12	0.46	202744.00	0.001	0.003
**Buffy**	Conc(Tape)	aTL_win	0.40	-0.03	0.70	1224.00	0.062	0.103
**PBMC**	DIN	aTL_win	0.33	0.11	0.53	48646.00	0.003	0.009
**PBMC**	%Unfrag	aTL_win	0.06	-0.17	0.29	71162.00	0.577	0.633
**PBMC**	%High Frag	aTL_win	-0.23	-0.43	0.00	93292.00	0.048	0.090

**PBMC**	%Severe Frag	aTL_win	-0.20	-0.41	0.03	91334.00	0.080	0.128
**PBMC**	A260/A280	aTL_win	0.16	-0.08	0.37	64260.00	0.177	0.242
**PBMC**	A260/A230	aTL_win	0.20	-0.03	0.41	60930.00	0.083	0.128
**PBMC**	Conc(Nano)	aTL_win	0.26	0.04	0.47	55928.00	0.020	0.045
**PBMC**	Conc(Pico)	aTL_win	0.16	-0.07	0.38	63768.00	0.160	0.225
**PBMC**	Conc(Tape)	aTL_win	0.19	-0.04	0.41	61352.00	0.092	0.138
