# Supplementary material for: Cross-tissue comparison of telomere length and quality metrics of DNA among individuals aged 8 to 70 years
Source: PLoS One. 2024 Feb 22;19(2):e0290918. doi: 10.1371/journal.pone.0290918 (PMC10883573; doi:10.1371/journal.pone.0290918)
Supplement: S10 Table — k = number of parameters in each candidate model, including the intercept; wi = Akaike model weight. Intercept-only and age-only null models are highlighted in gray for each tissue. (PDF) [file pone.0290918.s010.pdf]

Tissue aTL	Candidate Model Parameters	k	AICc	ΔAIC_c	w_i	R^2
Buccal	aTL ~ Age + %High Frag + Conc(Tape) + A260/A230 + 1	5	415.52	0.00	0.076	0.641
	aTL ~ Age + DIN + A260/A230 + 1	4	415.74	0.22	0.068	0.634
	aTL ~ Age + %High Frag + A260/A230 + 1	4	416.22	0.70	0.053	0.632
	aTL ~ Age + DIN + Sex + A260/A230 + 1	5	416.27	0.75	0.052	0.638
	aTL ~ Age + %High Frag + Sex + A260/A230 + 1	5	416.34	0.82	0.05	0.638
	aTL ~ Age + DIN + Race + A260/A230 + 1	6	416.5	0.98	0.046	0.643
	aTL ~ Age + %High Frag + Sex + Conc(Tape) + A260/A230 + 1	6	416.54	1.02	0.046	0.643
	aTL ~ Age + %High Frag + Race + Conc(Tape) + A260/A230 + 1	7	417.08	1.56	0.035	0.647
	aTL ~ Age + DIN + Conc(Tape) + A260/A230 + 1	5	417.18	1.66	0.033	0.635
	aTL ~ Age + %High Frag + Conc(Pico) + A260/A230 + 1	5	417.19	1.67	0.033	0.635
	aTL ~ Age + DIN + Race + Sex + A260/A230 + 1	7	417.49	1.97	0.028	0.645
	aTL ~ Age + 1	2	462.09	46.57	0	0.399
	aTL ~ 1	1	510.76	95.24	0	0
Saliva	aTL ~ Age + %Severe Frag + Race + 1	5	444.99	0.00	0.256	0.419
	aTL ~ Age + %Severe Frag + 1	3	446.19	1.20	0.141	0.387
	aTL ~ Age + %Severe Frag + Race + A260/A280 + 1	6	446.6	1.61	0.115	0.421
	aTL ~ Age + %Severe Frag + Race + Sex + 1	6	446.6	1.61	0.115	0.421
	aTL ~ Age + 1	2	486.35	41.36	0	0.027
	aTL ~ 1	1	486.73	41.74	0	0
DBS	aTL ~ Age + Race + Conc(Tape) + A260/A230 + 1	6	471.84	0.00	0.136	0.502
	aTL ~ Age + DIN + Race + Conc(Tape) + A260/A230 + 1	7	471.87	0.03	0.134	0.511
	aTL ~ Age + Conc(Tape) + A260/A230 + 1	4	472.47	0.63	0.099	0.48
	aTL ~ Age + DIN + Conc(Tape) + A260/A230 + 1	5	473.37	1.53	0.063	0.485
	aTL ~ Age + DIN + Conc(Tape) + A260/A280 + 1	5	473.54	1.70	0.058	0.484
	aTL ~ Age + 1	2	489.08	17.24	0	0.36
	aTL ~ 1	1	530.21	58.37	0	0
Buffy Coat	aTL ~ Conc(Nano) + 1	2	605.42	0.00	0.293	0.146
	aTL ~ Conc(Nano) + Sex + 1	3	606.26	0.84	0.192	0.154
	aTL ~ Age + Conc(Nano) + 1	3	607.32	1.90	0.113	0.147
	aTL ~ 1	1	622.42	17.00	0	0
	aTL ~ Age + 1	2	624.52	19.10	0	0
PBMC	aTL ~ Age + DIN + Sex + 1	4	355.53	0.00	0.1	0.307
	aTL ~ Age + Conc(Nano) + Sex + A260/A280 + 1	5	356.45	0.92	0.063	0.316
	aTL ~ Age + DIN + Sex + Conc(Tape) + 1	5	357.03	1.50	0.047	0.311
	aTL ~ Age + Conc(Nano) + %Unfrag + Sex + A260/A280 + 1	6	357.09	1.56	0.046	0.329
	aTL ~ Age + DIN + %Severe Frag + Sex + 1	5	357.14	1.61	0.045	0.31
	aTL ~ Age + DIN + Sex + A260/A280 + 1	5	357.37	1.84	0.04	0.308
	aTL ~ Age + 1	2	363.38	7.85	0.002	0.191
	aTL ~ 1	1	377.26	21.73	0	0
