# Supplementary material for: Cross-tissue comparison of telomere length and quality metrics of DNA among individuals aged 8 to 70 years
Source: PLoS One. 2024 Feb 22;19(2):e0290918. doi: 10.1371/journal.pone.0290918 (PMC10883573; doi:10.1371/journal.pone.0290918)
Supplement: S1 Fig — A datapoint was winsorized if it fell outside the range of (Q1-1.5IQR) to (Q3+1.5IQR) for its respective cohort-tissue distribution of data points, where Q1 and Q3 are lower and upper quartiles respectively, and the IQR is the interquartile ratio. Outlier values were winsorized to the boundary values of this range. 375/6673 (5.6%) datapoints were winsorized across the study. (PDF) [file pone.0290918.s011.pdf]

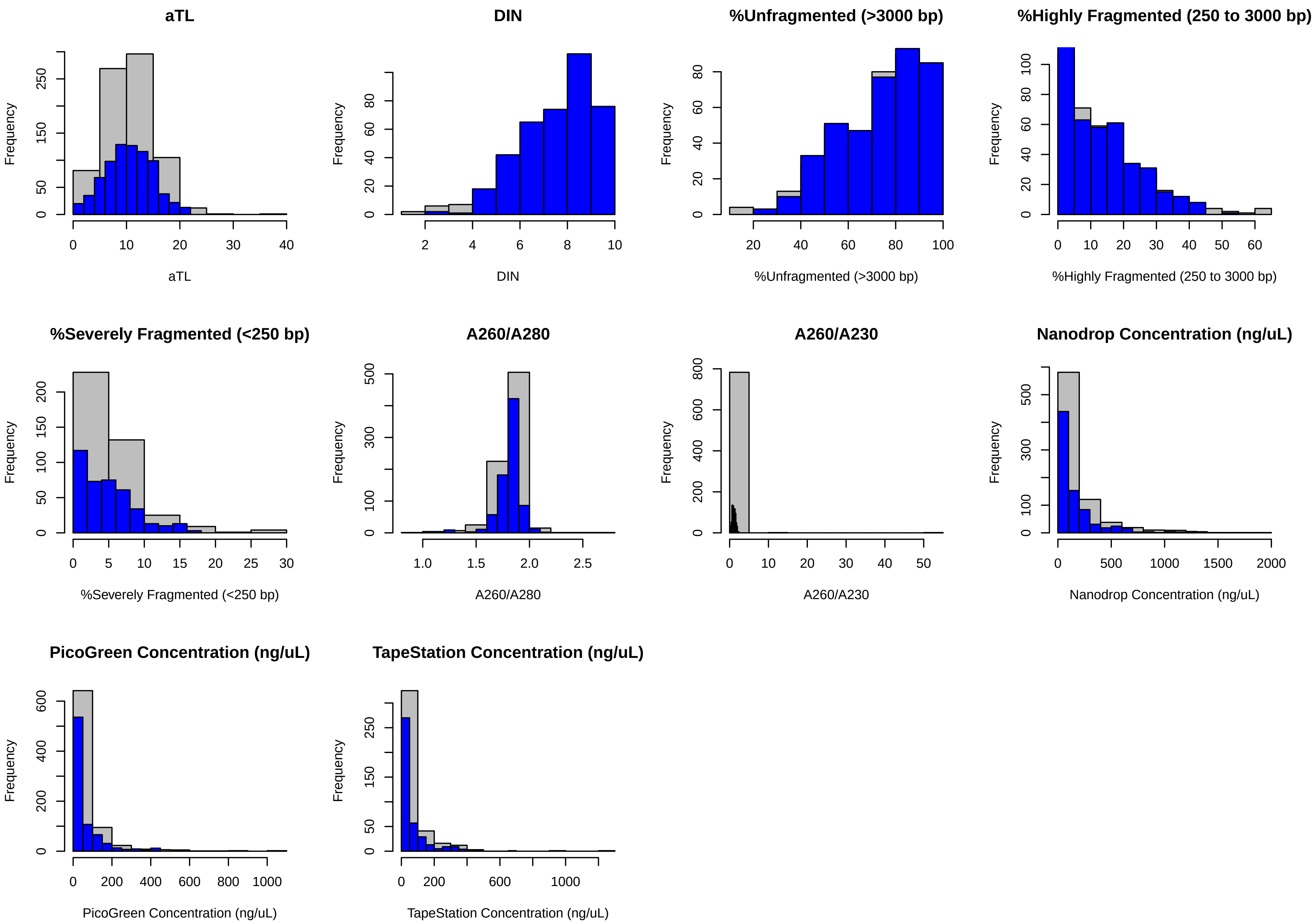

aTL
Frequency
aTL
DIN
Frequency
DIN
%Unfragmented (>3000 bp)
Frequency
%Unfragmented (>3000 bp)
%Highly Fragmented (250 to 3000 bp)
Frequency
%Highly Fragmented (250 to 3000 bp)
%Severely Fragmented (<250 bp)
Frequency
%Severely Fragmented (<250 bp)
A260/A280
Frequency
A260/A280
A260/A230
Frequency
A260/A230
Nanodrop Concentration (ng/uL)
Frequency
Nanodrop Concentration (ng/uL)
PicoGreen Concentration (ng/uL)
Frequency
PicoGreen Concentration (ng/uL)
TapeStation Concentration (ng/uL)
Frequency
TapeStation Concentration (ng/uL)
