# Supplementary figures and images for: Cross-tissue comparison of telomere length and quality metrics of DNA among individuals aged 8 to 70 years
Source: PLoS One. 2024 Feb 22;19(2):e0290918. doi: 10.1371/journal.pone.0290918 (PMC10883573; doi:10.1371/journal.pone.0290918)

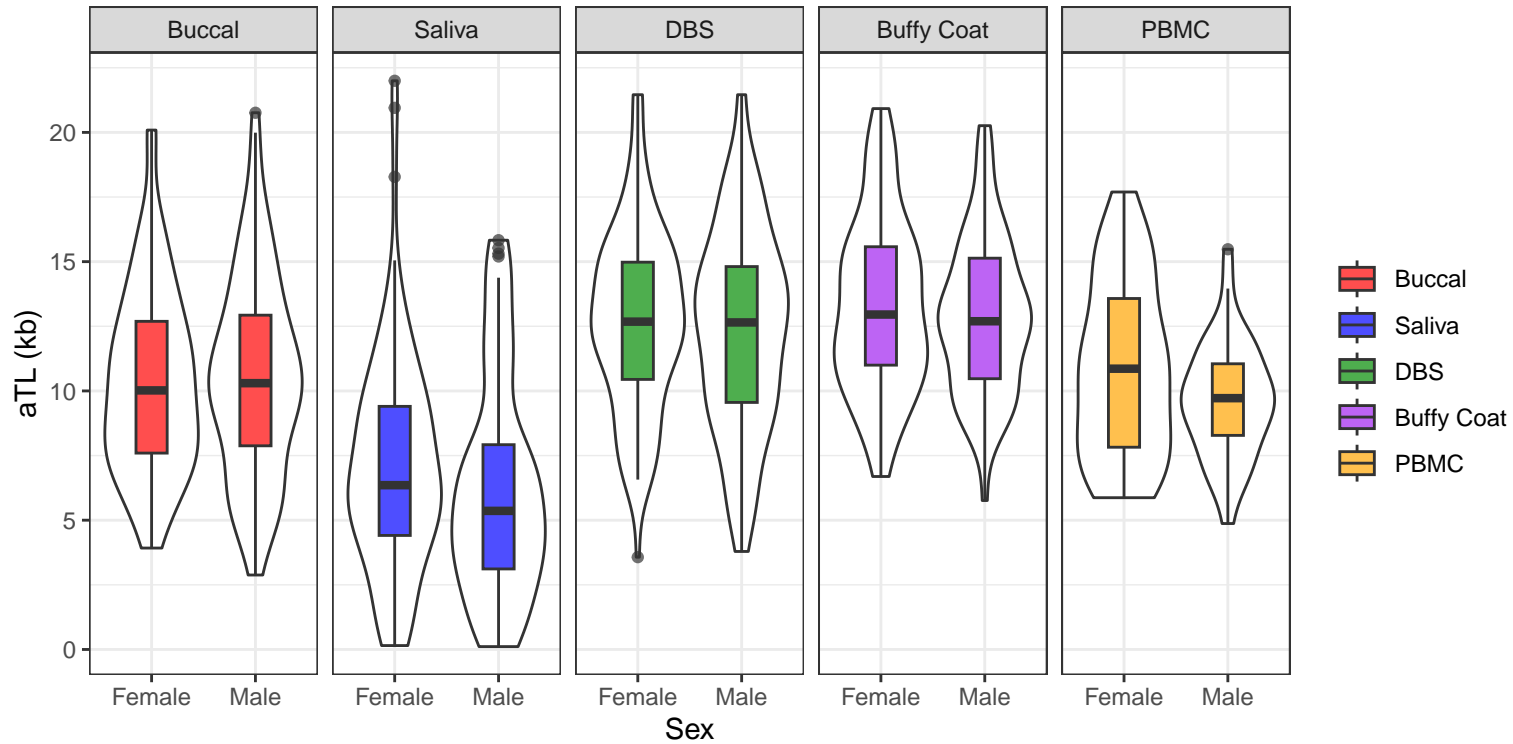

Supplement: S2 Fig — Buffy coat and PBMC are exclusive to child and adult cohorts, respectively. (PDF) [file pone.0290918.s012.pdf]

## Child Cohort

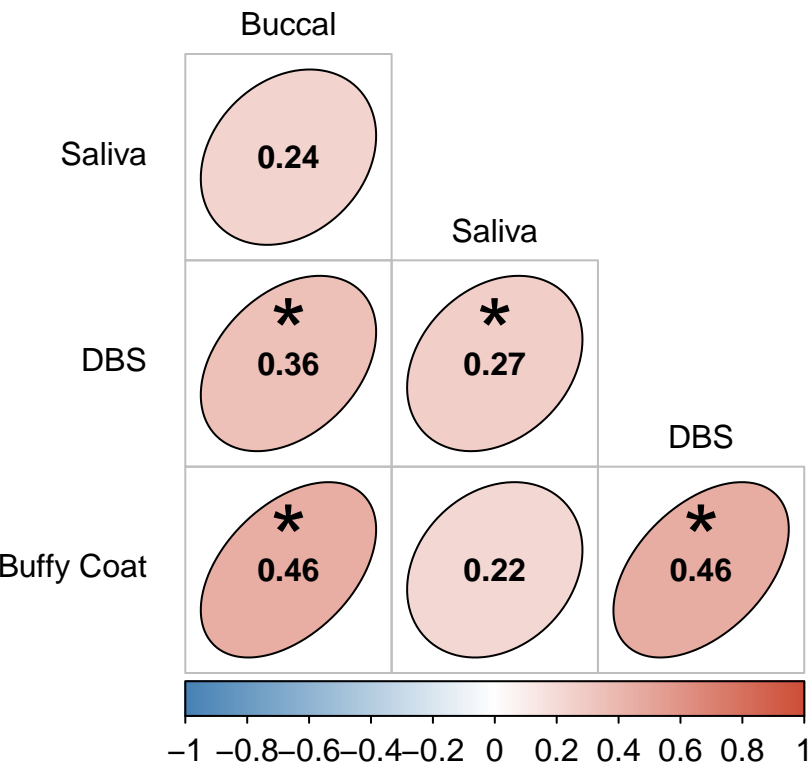

## Adult Cohort

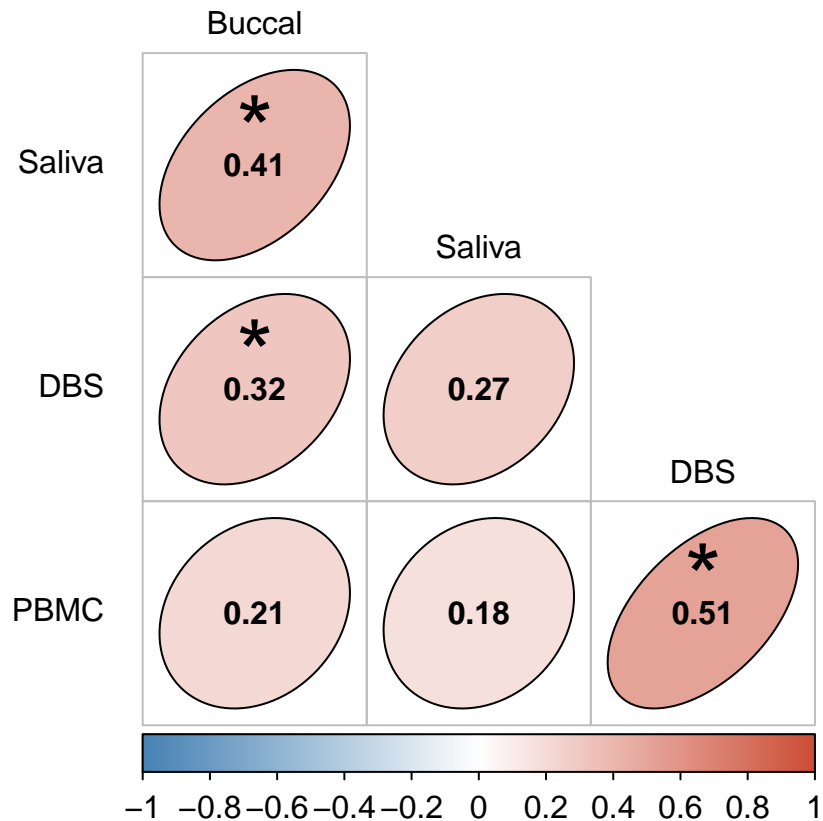

Supplement: S3 Fig — Ellipse shape and color denotes the strength and direction of correlations. Significant correlations (p < 0.05) are indicated by an asterisk. Buffy coat and PBMC are exclusive to the child or adult cohort, respectively. (PDF) [file pone.0290918.s013.pdf]

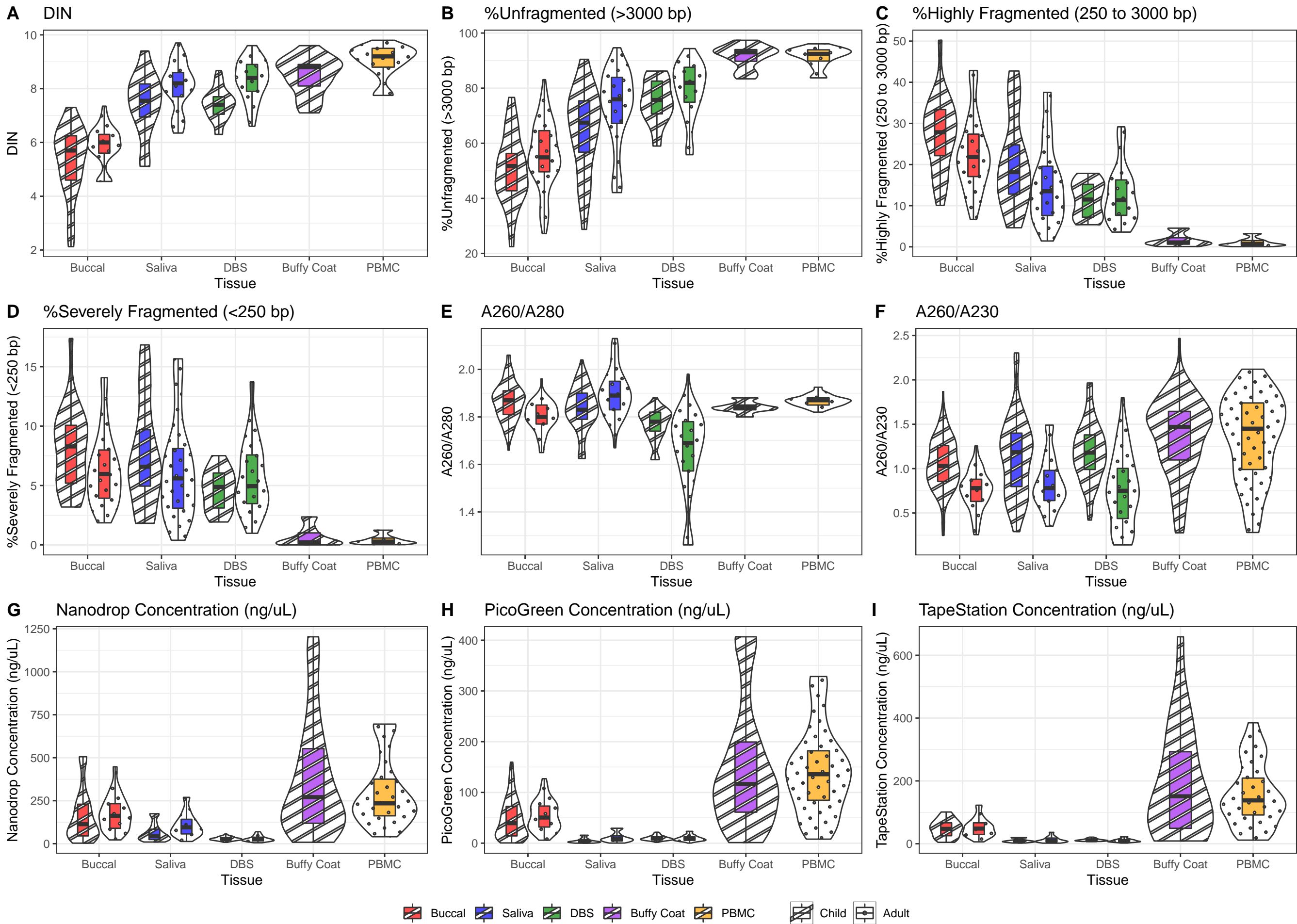

Supplement: S4 Fig — Buffy coat and PBMC are exclusive to child and adult cohorts, respectively. (PDF) [file pone.0290918.s014.pdf]

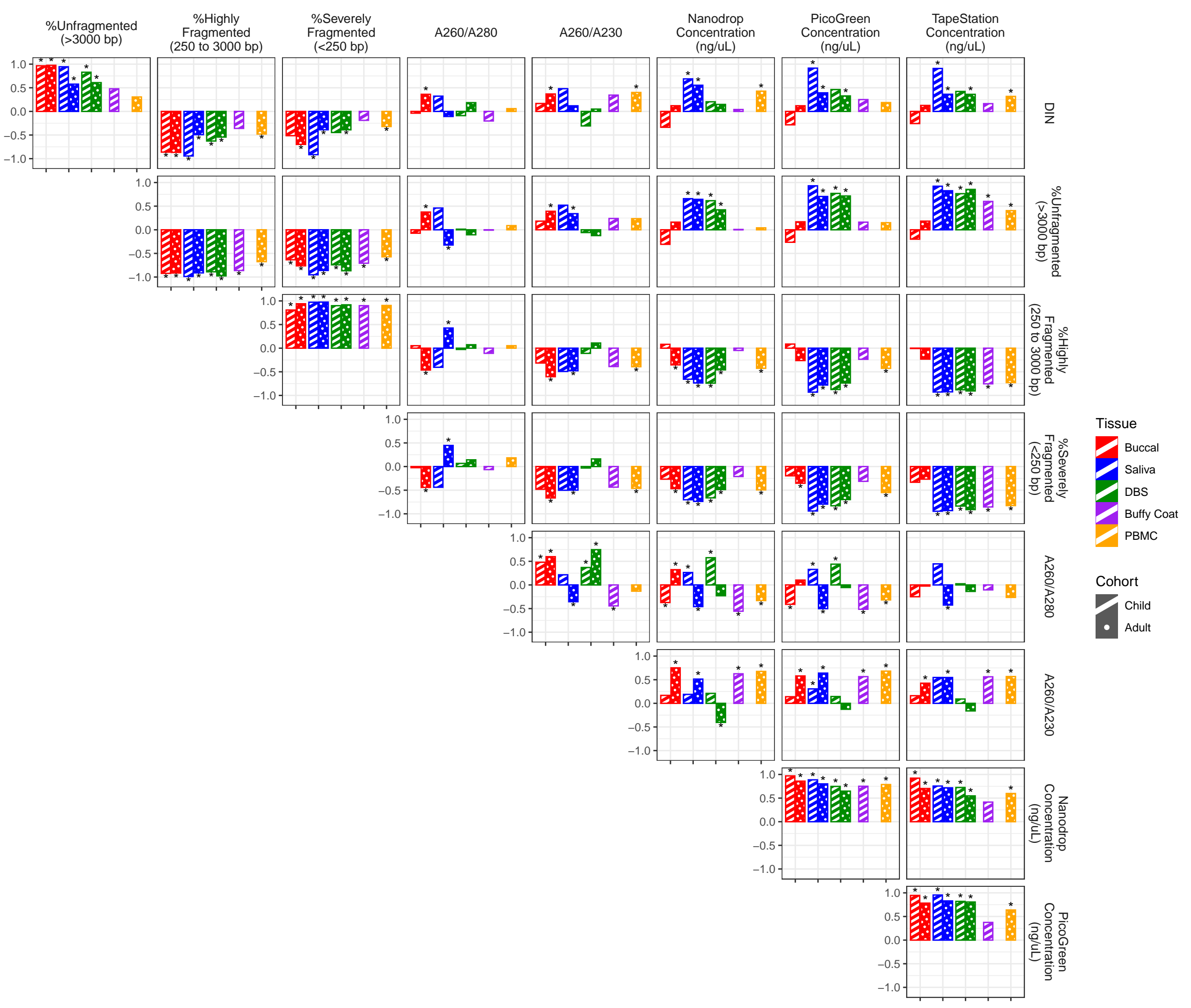

Supplement: S5 Fig — Y-axis p values range from -1 to 1, and significant correlations (p < 0.05) are indicated by an asterisk. (PDF) [file pone.0290918.s015.pdf]
