# Supplementary material for: Cross-tissue comparison of telomere length and quality metrics of DNA among individuals aged 8 to 70 years
Source: PLoS One. 2024 Feb 22;19(2):e0290918. doi: 10.1371/journal.pone.0290918 (PMC10883573; doi:10.1371/journal.pone.0290918)
Supplement: S6 Fig — (PDF) [file pone.0290918.s016.pdf]

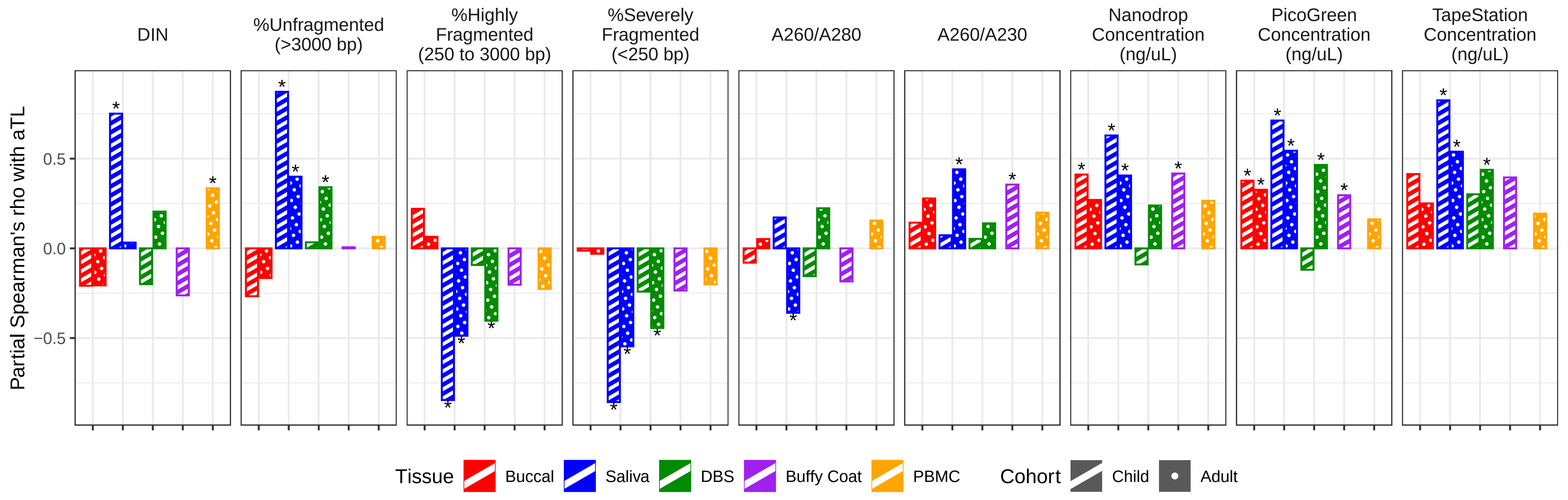

DIN
%Unfragmented (>3000 bp)
%Highly Fragmented (250 to 3000 bp)
%Severely Fragmented (<250 bp)
A260/A280
A260/A230
Nanodrop Concentration (ng/uL)
PicoGreen Concentration (ng/uL)
TapeStation Concentration (ng/uL)
Partial Spearman's rho with aTL
0.5
0.0
−0.5
Tissue
Buccal
Saliva
DBS
Buffy Coat
PBMC
Cohort
Child
Adult
