# Supplementary material for: Cross-tissue comparison of telomere length and quality metrics of DNA among individuals aged 8 to 70 years
Source: PLoS One. 2024 Feb 22;19(2):e0290918. doi: 10.1371/journal.pone.0290918 (PMC10883573; doi:10.1371/journal.pone.0290918)
Supplement: S7 Fig — Each panel shows the change in partial Pearson’s correlation (r) between aTL and age with the change of thresholds of quality DNA metrics for sample selection. “< =“ signifies keeping samples with quality DNA metrics of less than or equal to each value on the x-axis, and “> =“ signifies keeping samples with quality DNA metrics greater than or equal to each x value. The expectation is that removing samples will generally weaken r (become closer to zero), but removal based on certain DNA metric thresholds may lead to stronger and more negative age-aTL correlations. (PDF) [file pone.0290918.s017.pdf]

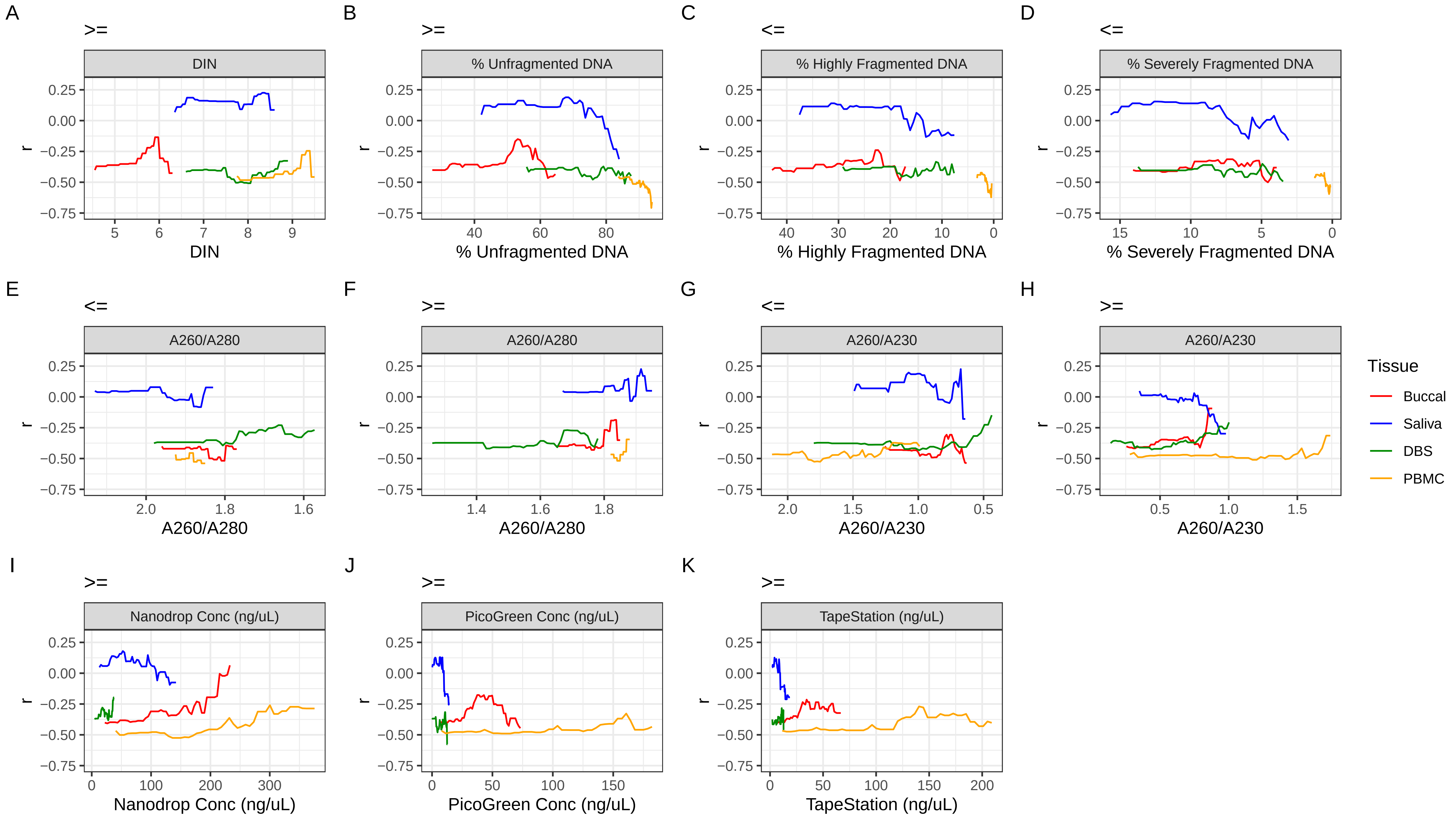

Only include adult samples;
r: Paritial Pearson's correlation between aTL and age after accounting for sex;
"<=" means keeping samples with DNA QC metrics of less than or equal to each value on the x axis;
">=" means keeping samples with DNA QC metrics of greater than or equal to each value on the x axis.
